# Can low-cost, scalable, online interventions increase youth informed political participation in electoral authoritarian contexts?

**DOI:** 10.1126/sciadv.adf1222

**Published:** 2023-06-28

**Authors:** Romain Ferrali, Guy Grossman, Horacio Larreguy

**Affiliations:** ^1^ Aix Marseille Univ, CNRS, AMSE, Marseille, France.; ^2^ University of Pennsylvania, Philadelphia, PA, USA.; ^3^ Instituto Tecnológico Autónomo de Mexico, Mexico City, México.

## Abstract

Young citizens vote at relatively low rates, which contributes to political parties de-prioritizing youth preferences. We analyze the effects of low-cost online interventions in encouraging young Moroccans to cast an informed vote in the 2021 elections. These interventions aim to reduce participation costs by providing information about the registration process and by highlighting the election’s stakes and the distance between respondents’ preferences and party platforms. Contrary to preregistered expectations, the interventions did not increase average turnout, yet exploratory analysis shows that the interventions designed to increase benefits did increase the turnout intention of uncertain baseline voters. Moreover, information about parties’ platforms increased support for the party closest to the respondents’ preferences, leading to better-informed voting. Results are consistent with motivated reasoning, which is surprising in a context with weak party institutionalization.

## Teaser

Scalabe online interventions may increase youth participation in electoral autocracies, with effects on vote choice.

## INTRODUCTION

Globally, youth electoral participation is low. Low youth turnout is likely consequential (*1*), as political parties tend to pursue policies that favor older citizens, who vote at higher rates (*2*, *3*). The reasons underlying such relatively low youth electoral turnout are a source of ongoing debates (*4*). It is possible that some youth want to engage in politics through voting but face high barriers to participating in the electoral process (*5*). In this case, lowering participation barriers should result in greater youth turnout. Alternatively, nonvoting youth may be uninterested in politics (*6*), or may not believe that elections are an effective means to bring about desired policy outcomes (*7*). In this case, lowering participation barriers is unlikely to result in greater turnout without structural changes to the political system. Whether lowering participation barriers can increase youth turnout is therefore an open question this paper helps address.

**Fig. 1.**
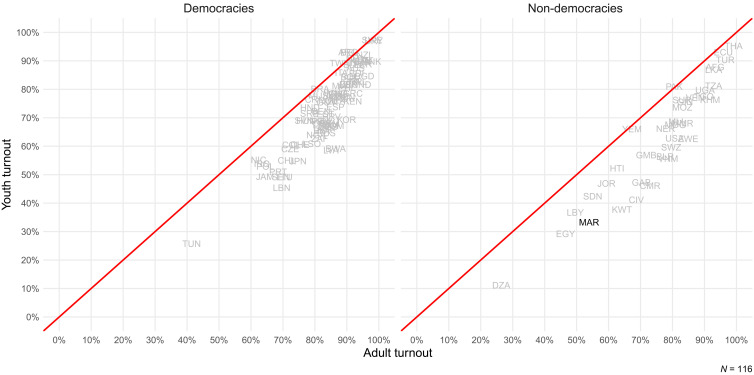
Turnout across the world. Younger citizens (*y* axis) have lower turnout rates compared to older citizens (*x* axis), especially in non-democracies (right). This figure reports survey estimates of youth (18 to 34) and adult (35+) turnout from the most recent election for which sufficiently high-quality data are available. Democracies are those countries that have a Polity-V score above 5. See section S1 for details about sources and construction.

We thus conduct a randomized controlled trial to test several scalable (i.e., able to reach a large number of voters at a relatively low cost)and theoretically grounded ways of encouraging youth turnout by lowering barriers to electoral participation. Against the backdrop of past work that mostly focuses on mature and young democracies, we test the effect of these low-cost interventions in the context of an electoral autocracy. Encouraging youth turnout is especially important in this setting, since the generational gap in voting is more pronounced in hybrid regimes that hold periodic and controlled elections (Fig. 1). Moreover, youth turnout is relevant because elections can improve policy congruence between citizens and public officials even in autocratic settings (*8*, *9*). Past work also suggests that high turnout improves the legitimacy of legislatures in autocratic settings (*10*). A more legitimate legislature that has an independent support base can play an important role in constraining autocrats (*11*) and promoting investment and economic growth (*12*, *13*).

Our study design is better understood using a simple Downsian model of voting (*14*), which we develop to guide and discipline exploratory (non-preregistered) subgroup analysis. Citizens vote (*y_i_* = 1) if their expected expressive utility from voting *u_i_* is larger than zero, and refrain from voting (*y_i_* = 0) otherwise

$$y_{i}=1\;⟺\;u_{i}=p^{*}+d-c>0$$

where (i) p* are the expressive policy benefits that accrue from voting for a party that would implement policies that are congruent with the citizen’s policy preferences, (ii) *d* are the expressive benefits associated with the civic duty of voting independent of vote choice, and (iii) *c* are the expected costs of voting. Our study’s three treatments, described below, match the parameters of this simple voting model.

First, it is possible that many young citizens do not turn out to vote because they perceive voting to carry too few benefits and too high a cost, in part due to knowledge gaps. Knowledge gaps increase the cost of voting (*c*) due to search costs (e.g., collecting information on how and where to register and/or vote) and decrease the perceived material benefits from voting (p*). Because party positions are not widely known, parties do not appear sufficiently different from each other to justify the cost of electoral participation. We thus preregistered that, on average, young citizens would be more likely to engage in electoral politics when they become more knowledgeable about the process of registration (*15*) and potential representatives' policy positions (*4*).

Second, especially in authoritarian settings, the youth lack direct experience of a functioning democracy, which has been shown to support the development and retention of norms and beliefs which strengthen electoral institutions (*16*). We thus preregistered that relaying basic civics information that highlights voting's role in strengthening electoral institutions would increase the benefit associated with the civic duty of voting independent of vote choice (*d*), thereby leading to an average increase in youth vote.

In collaboration with Tafra (https://tafra.ma), a Moroccan nonpartisan civil society organization, we designed a low-cost, scalable, locally appropriate, online informational campaign that aims at decreasing the perceived cost and increasing the expected benefit of voting. We studied the effects of Tafra’s campaign through a field experiment on the Moroccan youth. Given the importance of testing the effectiveness of civic interventions in hybrid regimes, Morocco was a good context for our purpose. Freedom House rates Morocco’s constitutional monarchy as “partly free” (*17*), youth participation is relatively low, and many voters are rather indifferent between parties (fig. S4), in part because most political parties are relatively young and not well institutionalized. Morocco’s legal framework allows for competitive legislative elections, but the transparency of the process is not guaranteed, and the King’s Palace continues to exert out-sized influence in the electoral process (*18*). Nonetheless, elections in Morocco are consequential for policy (*19*). Moreover, while the Ministry of Interior’s aggregated statistics indicate an average turnout of 42% in the 2016 general election, survey estimates reveal stark differences between the youth (33%) and older citizens (54%) (see section S1 for details about estimation).

Before the September 2021 general election, we recruited 7521 participants who were between the ages of 18 and 35, lived in Morocco, and received Facebook advertisements. Social media use is widespread in Morocco with Facebook being the most popular platform, as seen by 70.5% Moroccan internet users aged 16 to 64 using the platform monthly (*20*). Thus, in our setting, Facebook ads are an effective way to reach the population of interest. After a few questions on demographics, past and expected turnout, as well as party and policy preferences, study participants were randomly assigned to whether the online campaign (i) assisted them in checking their registration status on the voter file and, if necessary, assisted in navigating the registration process ("registration" treatment; designed to reduce *c*); (ii) provided them with civic education material, through a short video emphasizing the role of voting in strengthening institutions that aggregate citizen voice, and the alleged benefits of voting (“civics” treatment; designed to increase *d*); and (iii) helped them to identify the party most congruent to their policy preferences through an online tool that compared the stated policy positions of participants on a variety of politically salient issues to parties’ positions over those same issues. The tool showed treatment participants a list of parties ranked by increasing policy-preference distance to the respondent ("distance" treatment; designed to increase p*). After the treatments were delivered, participants were asked again about their expected turnout and party preferences. Moreover, after the election, we conducted a brief follow-up survey on actual turnout and party choice. While the distance and civics treatments also reduce search costs and hence *c*, we do not view this as a design flaw. Indeed, any exogenous dissemination of politically relevant information that affects *d* and p* will inadvertently also reduce *c*.

We developed our simple model to mainly guide and discipline our exploratory subgroup analysis, which we confirmed empirically using a causal forest approach (*21*) to show that the subgroups that emerge from theory are indeed the most important moderators of treatment effect (see Materials and Methods). Our online campaign need not affect the turnout choice of all study participants. Our approach extends a core insight of social context theory—namely, that political participation is subjected to a collective action problem (*22*, *23*)—and extends it to a Downsian model of turnout. Some citizens will always participate (“unconditional voters”), and some will never participate (“unconditional nonvoters”) even when faced with additional information on the potential and social benefit of voting and on the cost of not voting. These citizens have, respectively, high and low values of “net internal motivation” in the framework of Siegel (*24*). The lion’s share of the population, as demonstrated by Rolfe (*22*), are “conditional voters” who will choose to participate under certain conditions.

**Fig. 2.**
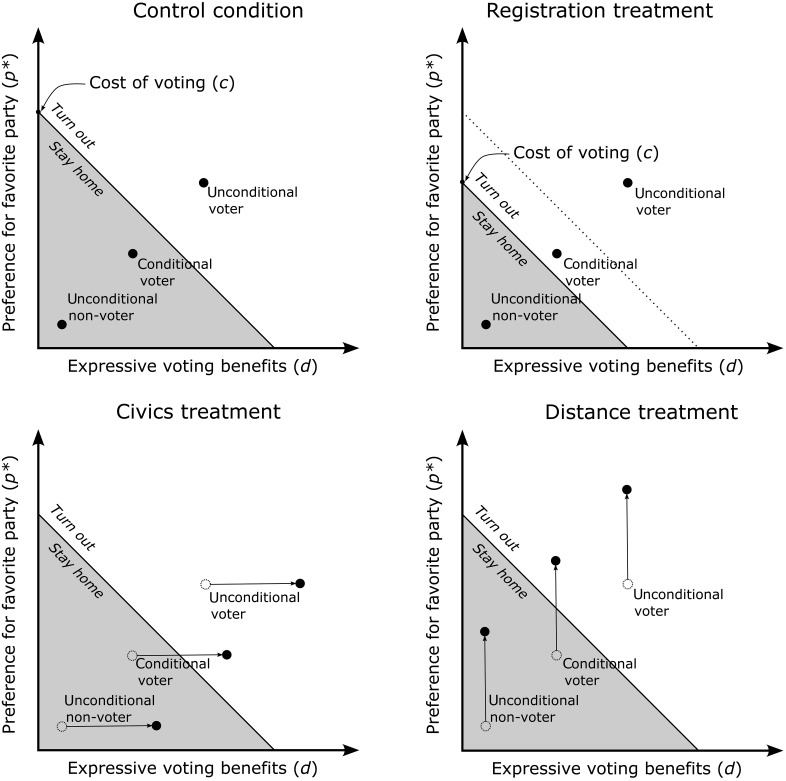
Illustration of the model’s theoretical implications to guide and discipline our exploratory subgroup analysis. The downward-sloping 45° line represents the set of points such that p* + *d* − *c* = 0; that is, such that the agent is indifferent between turning out to vote and refraining from voting for a fixed cost of voting *c*. The voter registration treatment is hypothesized to lower the cost of voting and, as such, to lower this indifference line (upper right panel).

Accordingly, in our exploratory subgroup analysis, we divided study participants into three subgroups: unconditional voters, who are committed to voting irrespective of their exposure to any additional messaging before elections, conditional voters, who do not turn out to vote in the status quo, and (unconditional) nonvoters, who will not vote even when exposed to any of the three treatments. This is because, for this group, relatively light-touch interventions cannot sufficiently increase *d* or p*, nor decrease *c* to push *u_i_* above zero. For example, this can be because those citizens hold a firm belief that the electoral process cannot generate meaningful benefits, or because their cost of voting is quite high. While our treatments cannot change the voting behavior of unconditional voters and nonvoters, they may do so for the third type of study participants: conditional voters who can be moved to turn out if exposed to a treatment. We preregistered that, on average, we should see a positive effect of treatments on turnout. Figure 2 summarizes the model’s theoretical implications by treatment, including our non-preregistered predictions on treatment heterogeneity by participant type.

Our main result is that, contrary to our preregistered hypothesis, none of the three treatments increased , intended youth turnout collected shortly after treatment delivery, self-reported after the election. We also report two tentative results that stem from exploratory analysis. First, we investigated heterogeneous effects by whether participants are conditional voters, nonvoters, or unconditional voters, based on voting intention elicited before treatment assignment (see the “Design and data” section). We find that both the civics treatment (designed to increase *d*) and the distance treatment (designed to increase p*) increased turnout intention for conditional voters only. The registration treatment had no discernible effect for any subgroup, presumably because implementation was somewhat convoluted as we were not allowed to interface directly with the state’s voter registration system [unlike, e.g., (*25*)].

Second, the distance treatment affected not only turnout—albeit only for conditional voters—but also voter choice. Consistent with our preregistered hypothesis, helping Moroccan youth assess their policy distance to the main political parties affected party preferences. After learning that their favorite party was not best aligned with their policy preferences, treatment voters were more likely to switch support toward a party more proximate to their policy preferences. In additional exploratory subgroup analysis, we further distinguished between those who had rated the party that the distance treatment deemed most congruent from a policy perspective as their second choice (thus exhibiting a small discrepancy between policy and party preferences) and those who had ranked that party third or lower (large discrepancy). Consistent with probabilistic voting models with voting costs (*26*), the effect is concentrated on participants exhibiting a small discrepancy. It is, furthermore, sizeable: about 20 percentage point reduction in actual voting for the party that participants had ranked as their favorite before treatment assignment. Moreover, contrary to our preregistered hypothesis, providing information on policy-preference distance to the main parties did not improve the stock of knowledge about those parties.

Motivated reasoning offers a plausible explanation for these findings. Strong versions of motivated reasoning hold that new information is processed in service of reaching a predetermined, desired conclusion (*27*); in other words, agents ignore information that goes against their prior beliefs. Weaker versions posit instead that agents ignore information that goes against their prior beliefs only when they can counter the credibility of the source on the basis of its accuracy (*28*) or reputation (*29*). In our case, citizens likely considered a signal consistent with their priors if it helped them rerank parties that they already ranked high: From the voter perspective, the tool made a subjectively plausible recommendation. By contrast, the signal was inconsistent with their priors if it suggested swapping the ranking of initially high- and low-ranked parties. In other words, respondents discarded information that went against their favorite party, in some, but not all cases, consistent with weaker versions of motivated reasoning. This behavior is inconsistent with Bayesian information processing. While Bayesian voters use their priors and new information to form beliefs about the accuracy of the information source, they use the prior beliefs, and not these updated beliefs, about the information source to interpret the new information (*30*).

That (weak) motivated reasoning seems to be at play in a setting with weakly institutionalized parties and low levels of partisan attachment is an important finding, as past work had suggested that in such settings, voters should be less likely to process information with bias. This explanation is bolstered by the fact that the median participant spent only about 16 s on the party distance treatment (fig. S5), suggesting that treated participants seemed keener on the tool’s “bottom line recommendation” rather than using it to educate themselves on the actual party positions in each policy domain. We reflect on the significance of this finding in the Discussion.

Our study contributes to the literature on the efficacy of civic education campaigns [(*31*, *32*) provide recent reviews]. Civic education is an important component of democracy promotion, but the evidence base in low- and middle-income countries is meager [for important exceptions, see (*33*–*36*)]. By showing that, while on average Tafra’s campaign had a null effect on turnout and vote intention, our simple model and exploratory subgroup analysis points to those who can be nudged to vote or to cast a more informed vote. In doing so, we build on work on motivated reasoning that cuts across various disciplines, including economics, political science, and psychology (*37*–*42*). In particular, our exploratory subgroup analysis suggests that young citizens, many of whom are relatively disengaged from politics, are also likely to engage in motivated reasoning. These findings not only inform theory, but also point to the need to incorporate this reaction in future civic education campaigns.

Moreover, departing from most existing work evaluating in-person, costly civic education campaigns [e.g., (*36*)], our study focuses on the effectiveness of low-cost, scalable, online interventions. The main exception is Finkel *et al.* (*43*), who use a similar recruitment method ahead of Tunisia’s 2019 presidential elections. Our studies, however, differ in the treatment content: Unlike Finkel *et al.*(*43*) and many past studies that focus on the benefits of democracy, Tafra’s campaign avoided this messaging because it was not deemed credible in Morocco’s non-democratic setting. Additionally, even if successful in the short run, such messaging could backfire in the longer run (*44*), if parties are unresponsive to new voters who, in turn, become further detached from politics.

We also enrich a burgeoning agenda that assesses empirically how to improve the quality or quantity of youth electoral engagement (*45*). We do so by focusing on online voter mobilization. Past studies that used social media platforms to increase electoral engagement generally do not target specifically the youth [e.g., (*46*, *47*)], which our theoretical framework and results suggest are likely to react differently to such campaigns. Finally, we contribute to research on electoral participation in non-democracies, which has mostly focused on either patronage linkages (*48*) or an urban-rural divide in participation (*49*), an education gap (*50*, *51*), and a gender divide (*52*). We depart from this literature by examining more proximate barriers to political participation and focusing on an important demographic group that receives too little scholarly attention: the youth.

### Design and data

We designed our study around Morocco’s local and general elections, which were held jointly on 8 September 2021. We conducted an online panel study around the election with the baseline survey conducted before the election and a follow-up survey conducted immediately after the election. To maximize the sample size, we used three rounds of recruitment spread between late June and early August 2021. For the sake of external validity and scalability of our results, we opted not to use a panel survey firm, and instead used Facebook ads to recruit participants. A total of 7521 participants completed the pre-election survey. Participation in the study was restricted to Moroccan citizens who were between 18 and 35 years old and currently resided in Morocco. Moroccan residency was confirmed by sending a confirmation text message to Moroccan-registered phone numbers. Those participants whose IP address was not registered in Morocco were further excluded from the sample. Since rounds 2 and 3 of the pre-election survey took place after the registration process had concluded, we further screened out citizens who were not registered on the voter file. All pre-election survey participants were recontacted via text messages after the election and invited to participate in the post-election survey (see section S2 for details about the survey flow and materials used).

In the pre-election survey, we collected respondents’ basic demographics, as well as their registration status. We further measured pretreatment levels of our study’s key outcomes: turnout intention and the identity of and level of support for respondents’ two most favorite parties. Participants also answered a series of questions in which we elicited their policy preferences on six politically salient issues on which political parties generally differ. For each of those questions, participants indicated (i) whether they agreed/disagreed/neither agreed nor disagreed with a specific policy, and (ii) whether such a policy was important to them (see table S3 for details about the policy questions used in the study).

During the pre-election survey, each respondent was randomly assigned to at most two of the following treatments. Table 1 provides details about the randomization.

**Table 1.** Number of participants by treatment group and study round. Percentages in parenthesis report, for each round, the percentage of participants assigned to each treatment condition.

	Baseline sample size	Treatment assignment						Endline retention rate, September 9 to October 9
		Control	Registration	Civics	Distance	Registration + distance	Civics + distance	
Round 1 *June 28 to July 3*	3615	951	879	0	924	861	0	12.1%
		(26.3%)	(24.3%)	–	(25.6%)	(23.8%)	–	
Round 2 *July 12 to July 18*	2022	495	0	508	504	0	515	7.9%
		(24.5%)	–	(25.1%)	(24.9%)	–	(25.5%)	
Round 3 *August 2 to August 8*	1884	448	0	490	468	0	478	5.5%
		(23.8%)	–	(26.0%)	(24.8%)	–	(25.4%)	

1. *Registration.* This module first verified whether respondents were registered on the voter file using the Ministry of Interior’s digital service. If respondents were registered, they were offered to verify their assigned polling station through the Ministry of Interior’s online service. If they were not registered, they were guided through the steps for registration using the same service. Study participants who were not assigned to this treatment condition were shown a placeholder that made them aware of the Ministry of Interior’s digital services. This treatment was implemented only in round 1, which took place in the last week of Morocco’s voter registration period.

2. *Civics*. Respondents assigned to this treatment were shown a 2-min civic education video that explained the responsibilities of the various bodies to be elected in the coming election and emphasized the importance of voting. Participants that were not assigned to this treatment were shown a placeholder that invited participants to “learn more about the upcoming elections” by browsing a third-party Instagram page.

3. *Distance*. Study participants assigned to this treatment were shown a screen that ranked the main Moroccan parties according to their policy distance to the respondent, including the value of such policy distance. We assigned a distance value of 1 for a given policy if the respondent’s preferences over that policy do not overlap with that of the party, and zero otherwise. The overall policy distance measure used equal weight for all policies that the respondent indicated as important, or for all six policies for which preferences were elicited if the respondent did not indicate any policy as important. If participants clicked over a particular party, they could see, for each of the six policies, whether the participant and the party had congruent policy views. The SI features details on the policy questions used and construction of policy distance (table S3) and a screenshot of the distance treatment interface (figs. S2 and S3).

Treatment assignment was stratified by round, registration status, prior likelihood to turn out to vote, and prior level of support for the respondents’ most favorite party. Table 1 provides information on the number of participants by treatment group and by study round, as well as on participation in the post-election survey. Note that the study faced high attrition (90.7%), which is not uncommon in the context of online panel surveys set in mid-income countries, where attrition rates often approach or exceed 60% [e.g., (*43*, *53*, *54*)]. In Materials and Methods, we discuss the implications of attrition for the robustness, statistical power, and validity of our findings.

## RESULTS

**Fig. 3.**
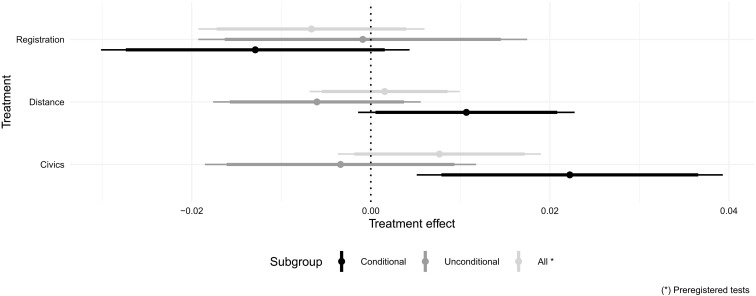
Treatment effects on turnout intention. This figure reports estimates from the models in tables S7 and S8. Bars are 90 and 95% heteroskedastic-robust confidence intervals.

Contrary to our preregistered hypotheses, the three treatments had, on average, no discernible effect on turnout intention, either in the short run (Fig. 3, sample “all”) or in the long run (table S7). However, our exploratory subgroup analysis suggests that there is heterogeneity consistent with the simple model we developed to guide such analysis. Figure 3 reports treatment effects on turnout intention in the pre-election survey broken down by treatment, and by whether participants are coded as likely “conditional” voters, or “unconditional” voters and nonvoters. We find that both the civics and distance treatments significantly increased the turnout intention of likely conditional voters by 2.3 and 1.1 percentage points on a 0 to 1 scale (*P* values < 0.05 and 0.1, respectively). These treatments, however, did not increase turnout intention for unconditional voters and nonvoters. Our ex-post power calculation shows that sample sizes in the short run are large enough to detect very small effects (Hedge’s *g* ≤ 0.2). We further use equivalence tests to show that effects on unconditional voters and nonvoters are indeed statistically indistinguishable from zero (see Materials and Methods).

The registration treatment, on the other hand, had no statistically significant effect on any subgroup, including conditional voters. We surmise that this is attributed to implementation difficulties related to this treatment: Navigation was somewhat convoluted because we were not authorized to interface directly with the Ministry of Interior’s digital service. That treatment had no significant effect on registration either (table S16). This outcome was, however, measured in the post-election survey where we faced severe power limitations, as we focused on participants who were not registered during the pre-election survey (*N* = 154).

While the civics and distance treatments increased voting intentions for conditional voters in the short run, we do not find evidence that these effects persisted in the long run, as self-reported in the post-election survey (table S8). Our long-term results are, however, only powered to detect, at best, small to medium effects [Hegde’s *g* ∈ (0.2,0.5)]; thus, this finding may reflect a genuine long-term null effect, or be an artifact of our smaller, self-selected sample (see Materials and Methods).

**Fig. 4.**
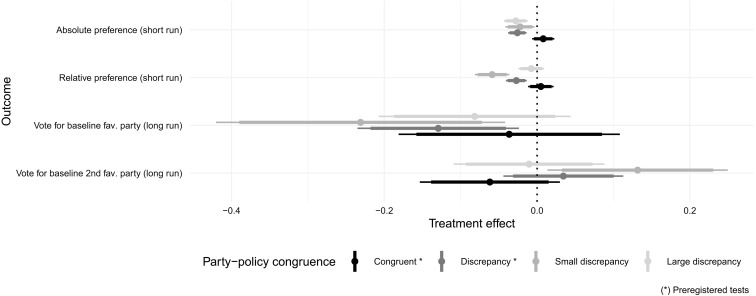
Effect of the distance treatment on party preferences (short-term outcomes measured in the pre-election survey) and voting decisions (long-term outcomes measured in the post-election survey). This figure reports estimates from models in tables S9 and S18. Bars are 90 and 95% heteroskedastic-robust confidence intervals.

Moving from turnout to vote choice, in Fig. 4, we present the results of our preregistered and exploratory analysis on the effect of the distance treatment on party preferences and ultimately voting decision, broken down by the extent of participants’ congruence between their policy and party preferences. The preregistered short-term results using the pre-election survey, first, indicate that those participants for whom the distance treatment revealed a (small or large) discrepancy reduced their absolute preference for their favorite party. This suggests that participants reduced their support for their favorite party when the distance treatment revealed that their policy preferences were not congruent with those of that party. Second, our exploratory subgroup analysis suggests that only those respondents for whom the distance treatment revealed a small discrepancy revised their relative preference for their most favorite party downward. In other words, treatment respondents only revised their relative preferences when learning that their second favorite party best matches their policy preferences. Similar to our analysis of turnout, we are sufficiently well powered to detect very small effects (Hegde’s *g* ≤ 0.2), and equivalence tests reveal that our null effects are indeed statistical zeroes (see Materials and Methods).

Turning to the long-run effects on vote choice using the post-election survey, our exploratory subgroup analysis suggests that participants for whom the distance treatment revealed a small discrepancy revised their likelihood of voting for their (pretreatment) favorite party downard, while conversely revising their likelihood of voting for their second favorite party upward, with seemingly large effect (23 and 13 percentage points, respectively). Revealing a large discrepancy led to no statistically significant revision in vote choice. These findings must be interpreted with care. As mentioned, recruitment into the post-election survey proved difficult, leaving us with a smaller, self-selected sample. In particular, we are only powered to detect, at best, small to medium effects [Hegde’s *g* ∈ (0.2,0.5)]. Moreover, the large long-term effects we detect for those with small discrepancies are also nonequivalent to zero (section S5.5.3). Consequently, while results are comparable to the short term, we cannot claim that the insignificant long-term effects we find for other subgroups are statistical zeroes. Materials and Methods provides the details of the robustness checks and ex-post power analysis we conducted to validate the vote choice results, including evidence that our vote choice long-term results are not artifacts of self-selection into the post-election survey.

With this caveat in mind, a form of motivated reasoning may explain these findings. Participants shown by the policy distance tool a large discrepancy may have found the information not credible enough to justify revising their vote choice. A series of results support this interpretation. Treated participants spent little time with the Tafra tool (16 s for the median participant; fig. S5). Coincidentally, and contrary to our preregistered hypothesis, the distance treatment did not lead to discernible improvements in knowledge of parties’ policy positions on average (table S10). Together, this suggests that participants mostly paid attention to the party ranking displayed in the main screen and compared it to their prior beliefs to assess whether it made sense, without paying much attention to the specifics underlying this ranking.

## DISCUSSION

We used a randomized controlled trial conducted ahead of Morocco’s 2021 general elections to test three interventions to increase youth electoral participation. We drew four conclusions from our preregistered and exploratory subgroup analyses.

Our main finding, namely, that all three preregistered treatments effects indicate a null average effect on turnout, suggests that we ought to be humble about the extent to which civil society groups can use relatively inexpensive mobilization strategies to improve electoral participation in authoritarian settings, especially where there is a relatively big reservoir of nonvoters who are disengaged from electoral politics (*50*). Such pattern has also been observed in Morocco, where some argue that many youth do not participate in the electoral process because they do not believe such process can bring about meaningful change [e.g., (*19*, *55*)].

Our remaining conclusions bear on exploratory analysis. First, results suggest it is nonetheless possible to use low-cost, scalable, online interventions to increase turnout of certain youth—specifically, those who are susceptible to changing their views on voting, i.e., conditional voters—by improving their expected benefit from participation through nudges about the value of voting or through the provision of relevant party-position information. Future interventions may usefully find ways to identify and target such conditional voters.

Second, our study also offers a cautionary tale about the ease of moving youth’s vote choice. On the one hand, the distance treatment improved congruence between policy and party preferences. On the other hand, the sizable effects we estimate for one subgroup—specifically those who had initially rated as second the party the distance treatment deemed as most congruent from a policy perspective—also suggest the possibility of a malleable electorate, potentially exposed to manipulations from nefarious actors spreading misinformation. Finding ways to assist citizens in recognizing the credibility of an online signalis an important avenue for future work (*56*).

Finally, our findings bear on theories of motivated reasoning, which maintain that individuals are more likely to be skeptical of information that threatens their prior beliefs (*39*). Results suggest that partisan-based motivated reasoning can be present even in settings where political parties are only weakly institutionalized. However, our study also suggests that many would accept and internalize information that results in updating against one’s favorite party, in particular when information helps respondents rerank parties that they already ranked relatively high. This qualification should be less likely in two-party systems, suggesting that the evidence of strong partisan-based motivated reasoning may simply be due to the fact that a large share of the current literature is US-focused.

## MATERIALS AND METHODS

### Ethics and preregistration

The study obtained ethics approval from the University of Pennsylvania’s and New York University Abu Dhabi’s Institutional Review Boards (IRB protocols #849223 and #HRPP-2021-63, respectively). Informed consent was secured online from all participants: All relevant information about the research was presented in text before the pre-election survey began. Participants had the opportunity to click on “agree to participate” before continuing. A pre-analysis plan for our study was preregistered at the egap Registry (https://osf.io/vypj7). Section S6 reports all our preregistered tests.

### Sample

We conducted our study with a sample of 7521 youth (18–35 years old) recruited using Facebook ads from June to August 2021. This sample is not perfectly representative of the Moroccan youth: among other demographics, it overrepresents male, educated, single individuals (table S4). As such, conclusions derived from this sample cannot be extended to the Moroccan population. However, the goal of the study was to speak precisely about the sample that can be reached over Facebook, whose use is widespread among the Moroccan youth. According to (*20*), in 2021, 22 million (78% of the population 13 years old or older) Moroccans were active social media users, and 19 million (67% of the population 13 years old or older) could be reached through Facebook ads. These users were also more likely to be male and young. These figures suggest that our sample is likely representative of the target population.

Our study design is composed of both a pre- and a post-election survey, with large attrition (90.7%) between the two waves. Those participating in the post-election survey were, on average, more educated and more interested in politics (table S4). We conducted a series of tests, described below, to verify that our results based on the post-election survey are not sensitive to such sample selection.

### Estimation

We derived the estimates presented above using ordinary least squares (OLS). These estimates allowed us to compare participants in the control condition to participants in the various treatment conditions. We made within-stratum comparisons, by including a stratum fixed effect. Let *Y_ij_* denote an outcome for individual *i* in stratum *j*, and $T_{i}^{c}\in \{0,1\}$ be a binary treatment indicator for treatment condition *c* ∈ {*C*(*ivics*), *D*(*istance*), *R*(*egistration*)}. Our first estimate, shown in Fig. 3, considers the average effect of our various treatments on turnout intention. It corresponds to the parameters *β_c_* in the following regression equation

$$Y_{ij}=\alpha_{j}+\beta_{C}T_{i}^{C}+\beta_{D}T_{i}^{D}+\beta_{R}T_{i}^{R}+\epsilon_{i}$$ (1)

with α*_j_* being a stratum fixed effect and ε*_i_* being a heteroskedastic-robust error term.

Figure 3 also amends the specification reported in Eq. 1 to estimate treatment effects by moderator. Let *M_i_* ∈ {0,1} be a binary variable that takes a value of 1 if individual *i* is a conditional voter and 0 otherwise—that is, if *i* is an unconditional voter or nonvoter. We estimate the following regression model

$$Y_{ij}=\alpha_{j}+\sum_{c\in \{C,D,R\}}\beta_{c}T_{i}^{c}+\sum_{c\in \{C,D,R\}}\gamma_{c}T_{i}^{c}M_{i}+\delta M_{i}+\epsilon_{i}$$ (2)

Figure 3 reports the parameter β*_c_* for the average effect of treatment *c* on nonvoters, as well as the parameters β*_c_* + γ*_c_* for the average effect of treatment *c* on conditional voters. Finally, Fig. 4 estimates a model comparable to the one in Eq. 2, with different outcomes, a different moderator (discrepancy), and removing parameters for the registration and civics treatments. This model additionally controls for prior relative support for one’s favorite party.

### Robustness

We conducted a series of additional tests. We first confirmed that our results on turnout hold when disaggregating unconditional voters and nonvoters into separate categories (table S11). In doing so, we ensured that our conclusion that no treatment increased the turnout of these two groups indeed applies to each group considered individually.

We then verified that only the distance treatment influenced party preferences. These placebo results (table S12) addressed the concern that the distance treatment simply increased the salience of policies, which led treated respondents to cast a vote that better capture the congruence between their policy preferences and that of the favorite political parties.

We then checked that attrition and selection cannot account for our results. We first show little evidence of differential attrition (fig. S8): while all treatments were associated with a comparable likelihood of participating in the post-election survey, the registration treatment was associated with a lower likelihood of completing the pre-election survey, confirming the implementation challenges of this treatment. We also checked that participants in the post-election survey have behavior that is comparable to that of attriters, by checking that our results derived from the pre-election survey generally travel to this admittedly selected sample (tables S7 to S9). Similarly, we checked that our results on the post-election survey would hold in the absence of attrition conditional on observables by reporting estimates weighted by the inverse probability of selecting into the post-election survey (section S5.3.2).

We then verified that our results are sufficiently well powered by conducting ex-post power calculations (section S5.3.3). We showed that the pre-election survey was sufficiently well powered to detect small effect sizes (Hedge’s *g* ≤ 0.2), while the post-election survey was only powered to detect medium to large effects (Hedge’s *g* > 0.2). We then tested whether our results are equivalent to zero. Our short-run results were nonambiguous: Statistically significant results were also not equivalent to zero, and statistically insignificant results were equivalent to zero. Our long-run results must, however, be interpreted with care: while our statistically significant results were not equivalent to zero, our statistically insignificant results were ambiguous, because they were too large to be equivalent to zero.

We finally verified that our moderators—that is, prior turnout intention and party-policy congruence—were the main drivers underlying heterogeneity in treatment effects and not simply picking up another underlying characteristic (section S5.4). This was especially important given that our subgroup analysis was not preregistered. Using the causal forest approach (*21*), we showed that prior turnout intention was the most important moderator of treatment effect for turnout intention among all the pretreatment characteristics we collected (fig. S9). Similarly, we considered absolute support for one’s favorite party and show that party-policy congruence is the second-most important moderator of the distance treatment (fig. S10).

## Supplementary Materials

### This PDF file includes:

Sections S1 to S6 Tables S1 to S26 Figs. S1 to S10 References
