# Supplementary material for: Can low-cost, scalable, online interventions increase youth informed political participation in electoral authoritarian contexts?
Source: Sci Adv. 2023 Jun 28;9(26):eadf1222. doi: 10.1126/sciadv.adf1222 (PMC10306298; doi:10.1126/sciadv.adf1222)
Supplement: Supplementary file 1 — Sections S1 to S6 Tables S1 to S26 Figs. S1 to S10 References [file sciadv.adf1222_sm.pdf]

Science Advances
AAAS

Supplementary Materials for

Can low-cost, scalable, online interventions increase youth informed political participation in electoral authoritarian contexts?

Romain Ferrali *et al.*

Corresponding author: Romain Ferrali, romain.ferrali@univ-amu.fr

Sci. Adv. **9**, eadf1222 (2023)
DOI: 10.1126/sciadv.adf1222

This PDF file includes:

Sections S1 to S6
Tables S1 to S26
Figs. S1 to S10
References

This document provides supplementary information for the manuscript "Can low-cost, scalable, online intervention increase youth informed political participation in electoral authoritarian contexts?". In section S1, we provide additional information on the construction of our motivating Figure 1. In section S2, we then provide further information on the study design and materials used to measure the main quantities of interest. In section S3, we provide descriptive statistics about our sample, as well as subsamples of interest. In section S4, we report the statistical models underlying our main results. In section S5, we report a series of tests we conducted to assess the robustness of our results. Finally, section S6 reports the tests pre-registered in the pre-analysis plan.

S1 Cross-country comparisons: Fig. 1

Fig. 1 reports estimated youth (18-34 years old) and adult (35+ years old) turnout during the last general election for which sufficiently high-quality data was available. We considered a large set of nationally representative surveys (628 surveys, for a total of more than 1m respondents) that featured the question "Did you vote in the election [the most recent national election, parliamentary or presidential] held in [year]?". Those surveys are enumerated in the table below:

Survey	Waves	Years	Initial N	Final N
AfroBarometer	1 to 7	1999 - 2018	250,287 (170)	57,454 (43)
Americas Barometer (LAPOP)	1 to 8	2004 - 2019	231,354 (135)	48,749 (28)
Arab Barometer	1 to 5	2006 - 2019	69,431 (47)	24,336 (15)
Asian Barometer	1 to 5	2001 - 2019	93,013 (61)	19,249 (12)
Comparative Study of Electoral Systems (CSES)	1 to 5	1996 - 2021	357,206 (215)	84,058 (51)
Total	-	-	1,001,291 (628)	233,846 (149)

Table S1. Surveys considered in the construction of Fig. 1. The columns "Initial N" and "Final N" indicate the number of respondents and number of nationally representative surveys (in parenthesis) considered initially and after the data filtering process, respectively.

Compiling these sources obtained a list of country surveys. Among this list, we first discarded those surveys for which there was uncertainty about the election year (e.g., when the survey partially overlaps with an election). We then discarded those respondents that were 17 years old or less during election year. We then pooled surveys covering the same election, and only considered those elections which featured (1) at least 250 responses for both youth and adults, and (2) overall non-response rates smaller than 10%. For each country-election, we derived youth and adult turnout rates for each survey using the available survey weights, and pooled the resulting survey estimates, weighting them by the resulting sample sizes. The procedure left us with 149 surveys, representing 233,846 respondents, covering 116 elections.

Finally, classification into democracies and non-democracies is based on Polity V scores. We classify as democracies those countries whose Polity V score is above 5 during the corresponding election year.

S2 Study design

S2.1 Study flow

Figure S1 below summarizes the flow of the survey experiment, including treatment manipulations.

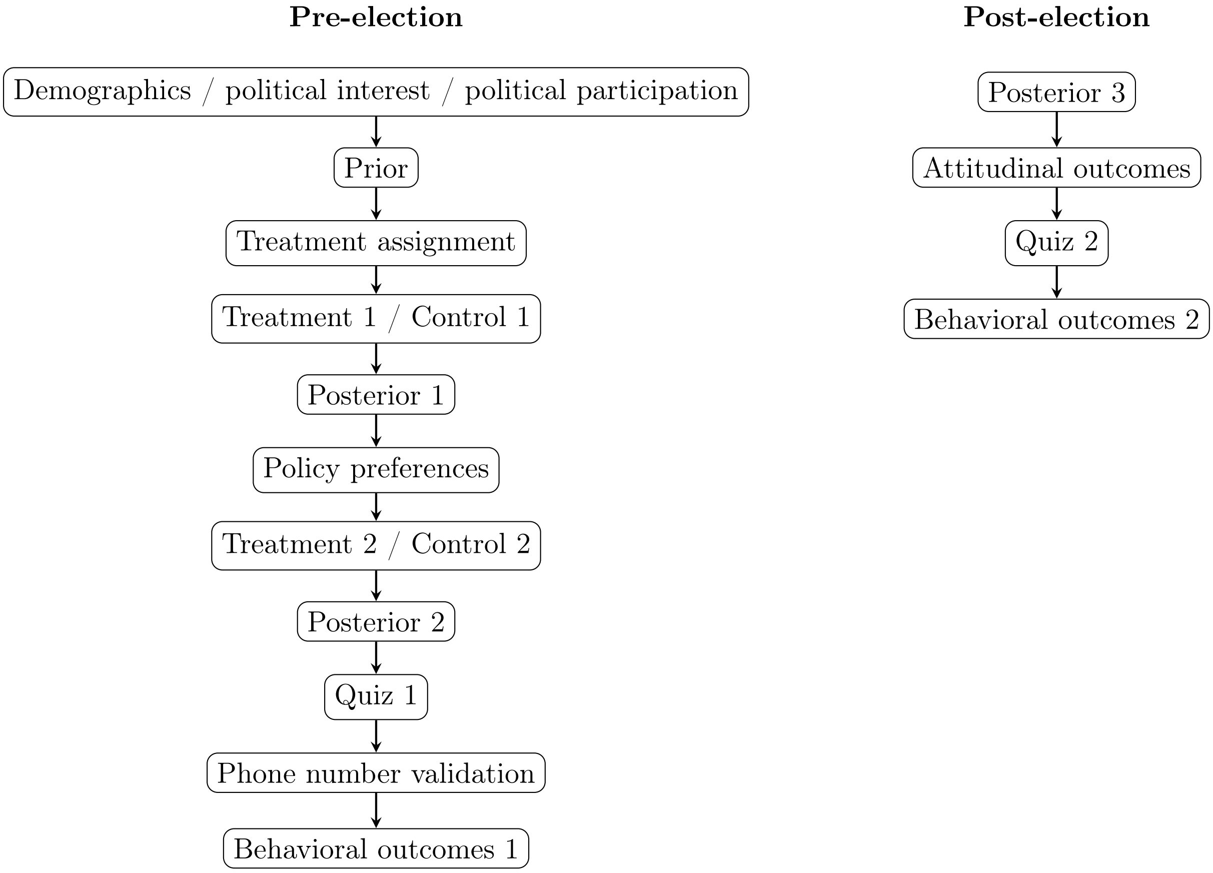

Fig. S1. Structure of pre- and post-election surveys. Our main results use data collected at "prior" and "policy preferences" to construct moderators, and outcomes measured at "posterior 2" and "quiz 1" (short-run outcomes) and "posterior 3" (long-run outcomes). "Treatment 1" corresponds to either the *registration* treatment (for round 1 of the baseline survey) or to the *civics* treatment (for rounds 2 and 3 of the baseline survey). "Treatment 2" corresponds to the *distance* treatment.

S2.2 Material

S2.2.1 Treatments and moderators

Table S2. Transcript of the video used in the *civics treatment*

English	Moroccan Arabic
On September 8, Morocco will hold three elections: legislative elections, regional elections, and communal elections.	فنهار 8 شتنبر غادي تدوز في المغرب تلاتا ديال الانتخابات الانتخابات التشريعية ، الإنتخابات الجهوية و الانتخابات الجماعية
During legislative elections, we choose, as Moroccan citizens, our representatives in Parliament, which will determine who will be the Head of Government, who is, as of today, Saadedine El Othmani.	فالانتخابات التشريعية كنختارو كمواطنين مغاربة النواب ديالنا في البرلمان و هادشي كيمكن من تحديد شكون غيكون رئيس الحكومة اللي هو حاليا سعد الدين العتماني.

MPs are those who make laws, monitor government activity, and evaluate public policies. Alongside with the government, they can also increase or reduce taxes, and decide upon the priorities (health, education, safety, etc.)	النواب البرلمانيون هما اللي كيوضعو القوانين ، كيراقبوا عمل الحكومة و كيديرو التقييم ديال السياسات العامة ويمكن لهم كدلك ، إلى جانب الحكومة ، على سبيل المثال : يزيدو فالضرائب أو يخفضوها ولا يقررو الأولويات (الصحة ، والتعليم ، والأمن ، وما إلى ذلك)
During regional elections, we elect our representatives in the regional council.	الإنتخابات الجهوية هي اللي كنتخبو فيها المتلين ديالنا في المجلس الجهوي
This body has important prerogatives, such as organizing transportation between cities, business support, and laying out tracks in rural areas, etc.	هاد الهيئة عندها صلاحيات مهمة بحال تنضيم النقل بين المدن دعم الشركات وإنجاز المسارات في المناطق القروية إلى آخره.
There are also communal elections, during which we choose our representatives in the communal council, which also determines who will be elected president of the commune – who is sometimes called the mayor.	و الانتخابات الجماعية، لي كنختارو فيها المثلين ديالنا في المجلس الجماعي، واللي كيمكن أيضا من انتخاب رئيس الجماعة اللي كيتسما فبعض الأحيان العمدة
Those are the representatives that are the closest to us. Their role is to manage our day-to-day public services: trash, markets, roads, water and electricity distribution, etc.	وهادو هما المنتخبين لي كيكونو قراب لينا كتر. كيتكلفو بتدبير المصالح العامة اليومية ديالنا : النفايات والأسواق والطرق وتوزيع المياه والكهرباء ، إلخ.
Every Moroccan citizen aged 18 or more has the right to vote in these elections.	كل مواطن مغربي تجاوز سن ديال 18 سنة عندو الحق في التصويت في كل من هذه الانتخابات.
In order to vote, I must be registered on the voter file, which will register me with a polling station.	باش نصوت خاصني نكون مسجل فاللوائح الانتخابية و بالتالي كنكون مسجل فمكتب التصويت.

On election day, I'll have to go to this polling station and bring my national ID. To find whether I'm registered and what is my polling station, I can send my national ID number to 2727, which is a phone service provided by the Ministry of Interior. I can also visit the website listeselectorales.ma	نهار التصويت خاصني نمشي لمكتب التصويت لي مسجل فيه و ندي معايا بطاقة التعريف الوطنية ديالي. باش نتأكد واش انا مسجل و فإينا مكتب التصويت نبعت رقم بطاقة التعريف الوطنية ديالي إلى ٢٧٢٧ ولي هي خدمة الهاتف التابعة لوزارة الداخلية ولا ندخل لموقع listeselectorales.ma
When I'll get to the polling station, the people in charge will explain to me how to vote. That's why I'll have to pay close attention to avoid making a mistake. Any mistake can make it so that my vote won't count.	مني غادي نوصل لمكتب التصويت، الناس لمكلفين غادي يشرحو لي طريقة التصويت ،هادشي علاش خصني نرد لبال مزيان باش منغلطش. أي غلط يقد يخلي ان التصويت ديالي مايتحسبش
Why not take a little bit of time to go to vote? That's my chance to choose who will govern the country, the region, or the city.	علاش مناخدش شوية دلوقت ونمشي نصوت ؟ هادي هي لفرصة باش نختار شكون غادي يسير البلاد ولاالجهةولا لمدينة
Voting is also our occasion to think about what happened in the previous period and to discuss what we want for our Morocco.	التصويت هو أيضا فرصة باش نفكرو ف اشنو طرى فالفترة لي دازت وناقشوا شنو بغينا للمغرب ديالنا
So, let's go vote on September 8?	إذن ، غادي نصوتو يوم 8 شتنبر ؟

Question	Parties						Sample	
	PAM	PI	PJD	PPS	RNI	USFP	Answer	Importance
Would you support allowing extramarital sexual relationships?	1	-1	-1	1	1	0	-0.286	0.819
Would you support that men and women be equal regarding inheritance?	1	-1	-1	1	-1	1	-0.369	0.819
As part of a tax reform, would you support that more Moroccans pay taxes?	-1	-1	-1	-1	1	1	-0.272	0.820
Would you support protectionist measures, such as increased tariffs to protect our domestic production, but also raise the price of imported goods?	1	-1	-1	1	-1	0	0.040	0.795
Would you support stopping subsidizing first necessity goods, such as gas cylinders?	-1	1	-1	-1	1	1	-0.428	0.838
Would you support that the state further encourages the development of private education and health?	1	-1	1	-1	-1	-1	0.084	0.852

Table S3. Computing policy distance. Columns refer to the parties used in the study. 1, -1, 0 indicate, respectively, the answers "Yes," "No," and "Maybe." Morocco counts 8 major parties (the 6 featured in the above table, as well as MP and UC). Party answers were collected from circulating a questionnaire containing 25 questions to the leadership of all 8 parties. Tafra completed missing data with available press statements. MP and UC were excluded from the analysis because not enough data could be found. From the remaining 6 parties, we removed questions with missing data, and questions whose answer was consensual (i.e., all parties gave the same answer). The procedure left the above 6 questions. We compute the policy distance $D_{ij} \in [0,1]$ between respondent i and party j by computing the percentage $P_{ij} \in [0,1]$ of policy preference questions for which i and j gave the same answer, among the questions that i found important. If i finds no question to be important, then we consider all questions asked to i. Policy distance is then $D_{ij} = 1 - P_{ij}$. Importantly, P_{ij} and D_{ij} are defined for all participants irrespective of treatment assignment. The *distance treatment* simply reveals P_{ij} to participants (see Fig. S2 and S3 for a screenshot).

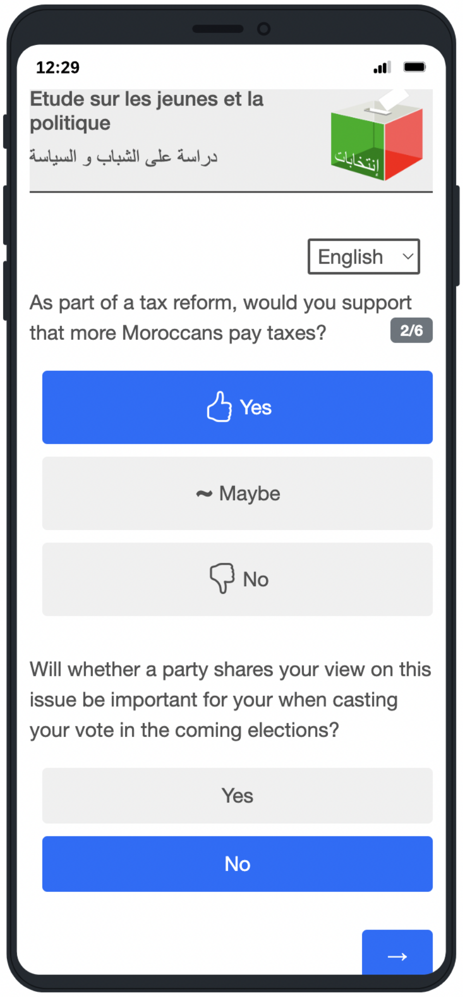

Fig. S2. Example policy preference question (mobile view)

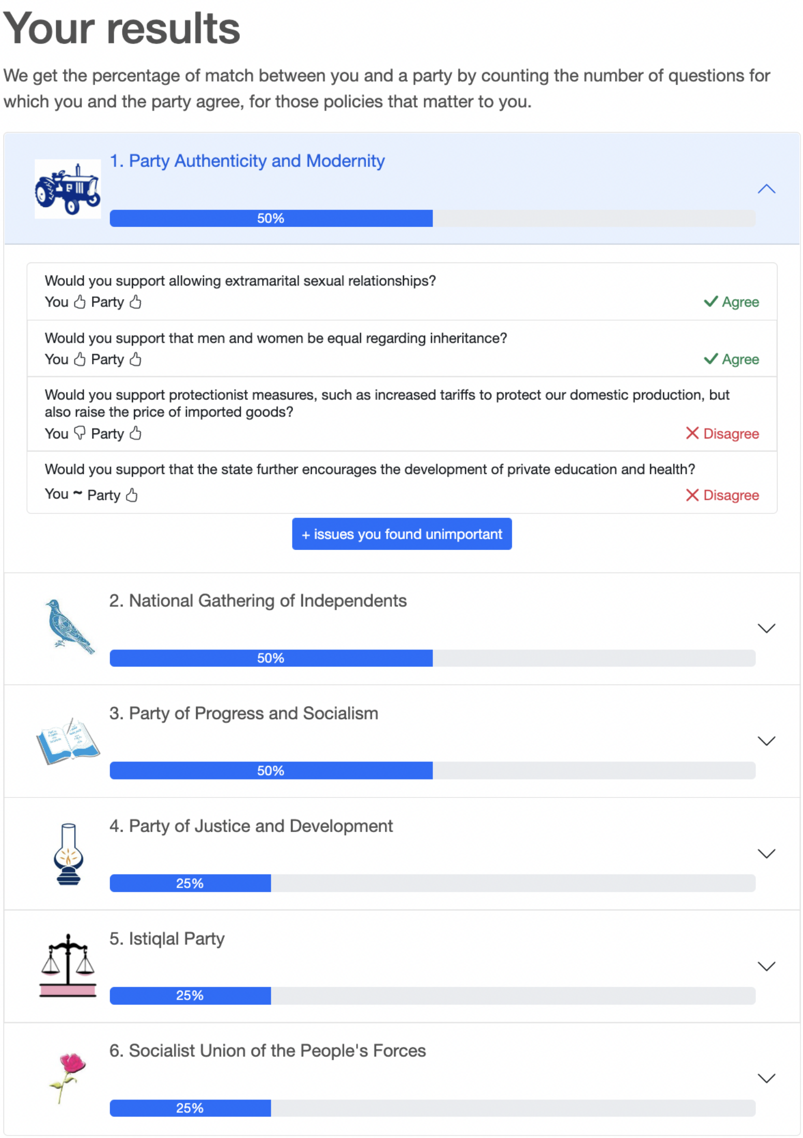

Fig. S3. Results page revealed by the distance treatment (desktop view)

S2.2.2 Outcomes

- Short-run outcomes (measured in the pre-election survey):
  - Turnout intention: measured using the following 5-points Likert scale, recoded to fall in the $[0, 1]$ range: "How likely is it that you will turn out to vote in the election that will be held on September 8 2021?" [Definitely not = 0 / Probably not / Not sure / Probably yes / Definitely yes = 1]
  - Level of support for one's favorite party: with $P_i^1, P_i^2 \in [0, 1]$ the level of support for i's favorite and second-favorite party respectively, we examine *absolute* support for one's favorite party P_i^1 as well as support *relative* to their second favorite party, $P_i^1 - P_i^2$. We measure support using the following 5-points Likert scale: "How close do you feel to those parties?" [List of participant's top two parties] [Very far = 0 / Somewhat far / Neither close nor far / Somewhat close / Very close = 1]
  - Political knowledge: measured as the number of correct answers to an incentivized quiz comprising 3 questions (see Quiz 1, Quiz 2 in Figure S1). The quiz picks 3 questions among the policy preferences (see Table S3) questions that the participant

found to be important, and asks for the answer that one of the participant's favorite two parties gave to the question. Questions are selected at random and, in the event that the participant chose fewer than three questions to be important, completed with questions the participant found unimportant. Parties are picked such that the participant's first favorite party appears twice, and their second favorite party appears once. This variable ranges from 0 to 3. Respondents earn one lottery ticket per correct answer. The lottery prize is a $10 gift card.

- Long-run outcomes (measured in the post-election survey):
  - Turnout: measured using the following question: "Did you turn out to vote in the elections that were held on September 8, 2021?" [No = 0 / Yes = 1]. The measure equals 0 for those participants who were not registered to vote. We also construct a measured of "inferred turnout," in which we use turnout intention (measured as in the pre-election survey) instead of turnout for those participants who were not registered to vote.
  - Vote choice: measured using the following question: "Please remember that this is an academic study. Your answers are anonymous and we will not share them with anyone. Which of the following parties did you vote for in the legislative election?" [Party of Justice and Development / Party Authenticity and Modernity / Istiqlal Party / National Gathering of Independents / Popular Movement / Socialist Union of Popular Forces / Party of Progress and Socialism / Constitutional Union / Other party / I cast a blank ballot / I would rather not say]. We recode participants' responses into a series of binary variables. The first such variable equals 1 if the respondent voted for their (pre-treatment) favorite party, and 0 otherwise; including if the respondent did not turn out to vote ("sharp" vote choice). Another version ("imputed" vote choice) uses the answer to the question "Had you voted in the legislative election, which of the following parties you would you have voted for?" in case the respondent did not turn out to vote. We further construct similar variables using participants' (pre-treatment) second favorite party instead of their first favorite party.
  - Political knowledge: measured as in the pre-election survey, and implemented with the same incentives.

S3 Additional descriptive statistics

S3.1 Sample, attrition, and treatment compliance

Variable	Sample	Population	Attriters	Non-attriters	Δ
**Socio-demographics**					
Age	24.501	-	24.499	24.522	0.024
% female	0.253	0.506	0.254	0.242	-0.012
% higher education	0.619	0.122	0.614	0.671	0.057***
% urban	0.675	0.611	0.672	0.703	0.031*
% Arabic	0.769	0.991	0.784	0.628	-0.156***
% single	0.787	0.563	0.785	0.811	0.026
% student	0.394	0.133	0.390	0.430	0.040**
% employed	0.234	0.407	0.234	0.225	-0.009
% IAM	0.520	-	0.513	0.583	0.070***
**Politics**					
Interest in politics[a]	0.479	0.710	0.478	0.483	0.005
% voted in 2016[a]	0.329	0.248	0.325	0.361	0.036*
% registered[b]	0.563	0.473	0.552	0.645	0.093***
Turnout intention (prior)	0.740	-	0.743	0.711	-0.032**
Attachment to party 1 (prior)	0.604	-	0.605	0.600	-0.004
% PJD supporters	0.319	-	0.321	0.297	-0.024
% RNI supporters	0.275	-	0.270	0.324	0.054***
**Design**					
Time spent on civics treatment	30.280	-	30.242	30.803	0.561
% compliers to civics treatment	0.167	-	0.165	0.186	0.020
Time spent on distance treatment	26.312	-	26.322	26.168	-0.154
% round 1 participants	0.481	-	0.466	0.623	0.157***
N	7521.000	-	6820.000	701.000	-

Note:
* p < .1, ** p < .05, *** p < .01.
[a] Population data: Arab Barometer wave 5 (2018)
[b] Among round 1 participants only, as being registered on the voter file was an eligibility criterion for rounds 2 and 3. Population data: Ministry of Interior (2021) aggregated data.

Table S4. Descriptive statistics. This table reports sample means for the whole sample ("Sample" column), as well as for attriters (i.e., respondents who did not participate in wave 2), and non-attriters (i.e., respondents who participated in wave 2). The "Population" column refers to population estimates. Those are derived from the 2014 census, unless otherwise mentioned. The Δ column reports the difference between attriters and non-attriters; p-values are robust. The variable % Arabic refers to the percentage of respondents that took the survey in Arabic. The variable % IAM refers to the percentage of respondents that use Itissalat Al Maghrib as their phone operator. The variables "Interest in politics", "Turnout intention (Prior)", "Attachment to party 1 (Prior)" are measured on a 0-1 scale, with 1 referring to the high modality. The variables "% PJD supporters" and "% RNI supporters" refer, respectively, to the share of respondents having chosen Party of Justice and Development, and National Gathering of Independents as their favorite party. The variable "% compliers to *civics treatment*" refers to the share of participants that have spent more than 60 seconds watching the civic education video.

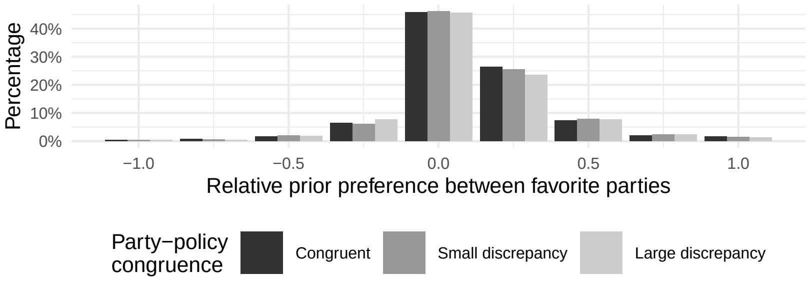

Fig. S4. Distribution of prior relative preference. This figure plots the distribution of participants' support for their favorite party relative to their second favorite party $(P_i^1 - P_i^2)$, by level of party-policy congruence. For all three levels of party-policy congruence, the bulk of participants have at most a weak preference for their favorite party (more than 60% of the mass in $[0, .25]$).

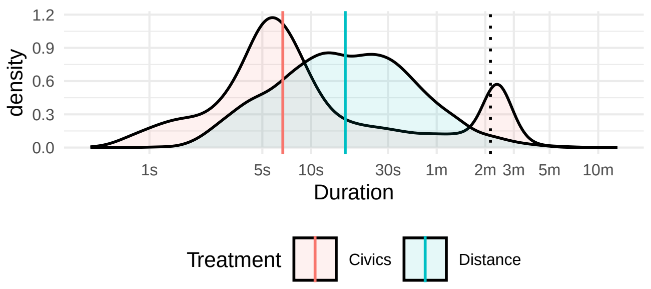

Fig. S5. Distribution of time spent on treatments. Colored lines are sample medians. The dotted line is the video's duration (2:09m). We report data from participants in rounds 2 and 3 only. Time spent on treatment was not recorded for round 1 and hence for the *registration treatment*. The median participant did not watch the civic education video (median time = 7s < 2:09m). The median participant spent 16 sec on the *distance treatment*.

S3.2 Moderators

S3.2.1 Prior turnout intention

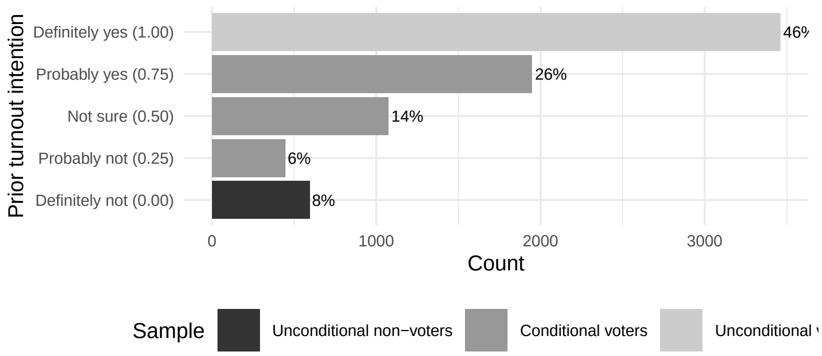

Fig. S6. Distribution of prior turnout intention. This figure provides a graphical representation of possible answers to the question: "How likely is it that you will turn out to vote in the election that will be held on September 8 2021?". 48% participants are likely conditional voters.

Variable	Prior turnout intention: Uncond. non-voters	Cond. voters	Uncond. voters	Differences: Δ (Cond. voters)	Δ (Uncond. voters)
**Socio-demographics**					
Age	25.550	24.256	24.565	-1.293***	-0.984***
% female	0.201	0.271	0.245	0.070***	0.044**
% higher education	0.638	0.621	0.614	-0.017	-0.024
% urban	0.706	0.692	0.653	-0.014	-0.053***
% Arabic	0.736	0.732	0.812	-0.004	0.076***
% single	0.776	0.810	0.766	0.034*	-0.010
% student	0.339	0.411	0.387	0.072***	0.048**
% employed	0.284	0.207	0.251	-0.077***	-0.033
% IAM	0.496	0.527	0.517	0.031	0.021
**Politics**					
Interest in politics[a]	0.346	0.391	0.589	0.045***	0.243***
% voted in 2016[a]	0.316	0.252	0.407	-0.064***	0.091***
% registered[b]	0.358	0.419	0.783	0.061**	0.425***
Turnout intention (prior)	0.000	0.608	1.000	0.608***	1.000?
Attachment to party 1 (prior)	0.344	0.534	0.714	0.190***	0.369***
% PJD supporters	0.328	0.336	0.300	0.009	-0.028
% RNI supporters	0.202	0.273	0.290	0.071***	0.088***
**Design**					
Time spent on civics treatment	27.612	29.418	31.342	1.806	3.730
% compliers to civics treatment	0.121	0.163	0.175	0.042*	0.055**
Time spent on distance treatment	30.463	27.625	24.679	-2.838	-5.784*
% round 1 participants	0.610	0.508	0.431	-0.102***	-0.179***
N	595.000	3467.000	3459.000	-	-

Note:
* $p < .1$, ** $p < .05$, *** $p < .01$.

Table S5. Descriptive statistics, by prior turnout intention. This Table uses the same conventions as Table S4 but breaks the sample down as a function of their prior turnout intention. The Δ columns compare the column in parenthesis to the "Unconditional non-voters" column.

S3.2.2 Party-policy congruence

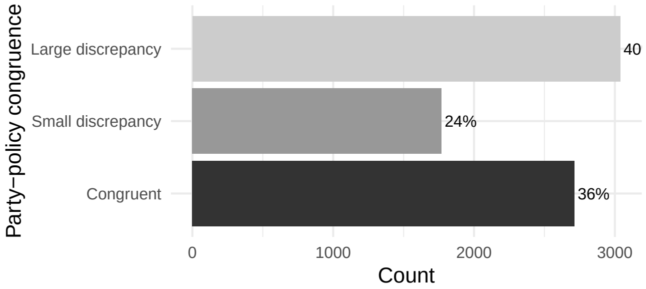

Fig. S7. Distribution of party-policy congruence

Variable	Party-policy congruence: Congruent	Small discrepancy	Large discrepancy	Differences: Δ (small)	Δ (large)
**Socio-demographics**					
Age	24.513	24.308	24.603	-0.205	0.090
% female	0.270	0.240	0.246	-0.030**	-0.025**
% higher education	0.636	0.604	0.613	-0.032**	-0.023*
% urban	0.690	0.671	0.664	-0.019	-0.026**
% Arabic	0.781	0.774	0.755	-0.007	-0.026**
% single	0.773	0.813	0.785	0.040***	0.012
% student	0.397	0.413	0.380	0.016	-0.017
% employed	0.226	0.239	0.237	0.013	0.011
% IAM	0.521	0.534	0.510	0.012	-0.011
**Politics**					
Interest in politics[a]	0.475	0.476	0.483	0.001	0.008
% voted in 2016[a]	0.326	0.324	0.334	-0.002	0.008
% registered[b]	0.530	0.574	0.585	0.044**	0.055***
Turnout intention (prior)	0.738	0.740	0.743	0.001	0.004
Attachment to party 1 (prior)	0.609	0.596	0.605	-0.013	-0.005
% PJD supporters	0.533	0.213	0.189	-0.320***	-0.344***
% RNI supporters	0.133	0.367	0.348	0.235***	0.216***
**Design**					
Time spent on civics treatment	30.459	30.816	29.783	0.357	-0.677
% compliers to civics treatment	0.167	0.168	0.165	0.001	-0.002
Time spent on distance treatment	23.933	27.754	27.698	3.821**	3.765**
% round 1 participants	0.464	0.475	0.499	0.011	0.035***
N	2713.000	1769.000	3039.000	-	-

Note:
* p < .1, ** p < .05, *** p < .01.

Table S6. Descriptive statistics, by congruence between party preferences and policy preferences. This Table uses the same conventions as Table S4 but breaks the sample down as a function of their congruence. The Δ columns compare the column in parenthesis to the "Congruent" column.

S4 Main results

	Pre-election: All	Pre-election: Non-attriters	Post-election: Turnout	Post-election: Inferred turnout
registration	−0.007	−0.008	−0.029	−0.015
	(0.006)	(0.019)	(0.046)	(0.042)
civics	0.008	−0.020	0.012	0.012
	(0.006)	(0.025)	(0.060)	(0.060)
distance	0.002	−0.018	−0.035	−0.014
	(0.004)	(0.016)	(0.036)	(0.034)
Num.Obs.	7521	701	700	700
R2	0.656	0.756	0.374	0.289
Mean DV (Control)	0.753	0.760	0.672	0.746
Min. DV	0	0	0	0
Max. DV	1	1	1	1

Table S7. Average treatment effects on turnout. This table reports OLS estimates corresponding to the model in equation 1. All models include stratum-fixed effects. Standard errors are robust to heteroskedasticity. See section S2.2.2 for a definition of outcomes. The model in column 1 is used to construct Figure 3 in the manuscript. No treatment had a statistically significant short-run average effect on turnout (column 1). The finding also applies to non-attriters (column 2). Treatments had no significant long-term effects (columns 3 and 4). * p < .1; ** p < .05; *** p < .01.

	Pre-election		Post-election	
	All	Non-attriters	Turnout	Inferred turnout
registration	−0.0009	−0.012	−0.026	−0.044
	(0.009)	(0.026)	(0.059)	(0.059)
civics	−0.003	−0.043	−0.019	−0.019
	(0.008)	(0.032)	(0.077)	(0.077)
distance	−0.006	−0.045*	−0.046	−0.028
	(0.006)	(0.023)	(0.046)	(0.046)
registration × conditional	−0.012	0.011	−0.006	0.066
	(0.013)	(0.038)	(0.095)	(0.085)
civics × conditional	0.026**	0.047	0.065	0.064
	(0.012)	(0.050)	(0.121)	(0.120)
distance × conditional	0.017*	0.058*	0.022	0.029
	(0.009)	(0.031)	(0.074)	(0.069)
Num.Obs.	7521	701	700	700
R2	0.656	0.759	0.374	0.290
Mean DV (Control, unconditional)	0.857	0.879	0.770	0.813
Mean DV (Control, conditional)	0.633	0.648	0.581	0.683
Min. DV	0	0	0	0
Max. DV	1	1	1	1
distance + distance × conditional	0.011*	0.014	−0.023	0.001
	(0.084)	(0.501)	(0.686)	(0.978)
registration + registration × conditional	−0.013	−0.001	−0.032	0.021
	(0.142)	(0.971)	(0.666)	(0.725)
civics + civics × conditional	0.022**	0.004	0.046	0.046
	(0.011)	(0.914)	(0.616)	(0.621)

Table S8. Average treatment effects on turnout, by prior vote intention. This table reports OLS estimates corresponding to the model in equation 2. All models include stratum-fixed effects. Standard errors are robust to heteroskedasticity. See section S2.2.2 for a definition of outcomes. The bottom panel reports the linear combination of parameters reported in each row. The p-value associated with the corresponding F-test is reported in parentheses. The model in column 1 is used to construct Figure 3 in the manuscript. The *distance* and *registration treatments* significantly increased turnout in the short run for conditional voters only (column 1). Non-attriters show somewhat comparable patterns (column 2). Treatments had no significant long-term effects (columns 3 and 4). * $p < .1$; ** $p < .05$; *** $p < .01$.

	Pre-election				Post-election			
	Abs. pref		Rel. pref		Vote for fav.		Vote for 2nd fav.	
	All	Non attr.	All	Non attr.	Sharp	Imputed	Sharp	Imputed
distance	0.008	0.068**	0.005	0.058	−0.037	0.013	−0.062	−0.048
	(0.007)	(0.027)	(0.008)	(0.035)	(0.074)	(0.083)	(0.047)	(0.057)
small discrepancy	−0.017**	0.055**	−0.021**	0.027	0.218**	0.261***	−0.069*	−0.065
	(0.008)	(0.026)	(0.008)	(0.031)	(0.090)	(0.092)	(0.042)	(0.054)
large discrepancy	−0.007	0.023	−0.009	0.028	0.060	0.081	0.035	0.074
	(0.007)	(0.023)	(0.008)	(0.029)	(0.068)	(0.074)	(0.051)	(0.062)
distance × small discrepancy	−0.030**	−0.095*	−0.064***	−0.187***	−0.194	−0.295**	0.193**	0.202**
	(0.012)	(0.049)	(0.014)	(0.057)	(0.121)	(0.129)	(0.077)	(0.089)
distance × large discrepancy	−0.036***	−0.096**	−0.013	−0.049	−0.045	−0.095	0.051	0.013
	(0.011)	(0.038)	(0.012)	(0.045)	(0.098)	(0.109)	(0.069)	(0.082)
prior	−0.064***	−0.060	0.687***	0.687***	0.001	0.039	−0.126**	−0.076
	(0.012)	(0.049)	(0.017)	(0.068)	(0.097)	(0.106)	(0.060)	(0.072)
Num.Obs.	6825	630	6757	625	551	551	551	551
R2	0.636	0.680	0.443	0.566	0.267	0.219	0.197	0.209
Mean DV (Control, no discrepancy)	0.642	0.588	0.129	0.096	0.226	0.313	0.104	0.157
Mean DV (Control, small discrepancy)	0.613	0.656	0.102	0.143	0.510	0.612	0.041	0.082
Mean DV (Control, large discrepancy)	0.629	0.623	0.112	0.104	0.316	0.395	0.140	0.202
Min. DV	0	0	−1	−1	0	0	0	0
Max. DV	1	1	1	1	1	1	1	1
distance + distance × small discrepancy	−0.022**	−0.027	−0.059***	−0.129***	−0.231**	−0.282***	0.132**	0.154**
	(0.023)	(0.510)	(0.000)	(0.004)	(0.017)	(0.005)	(0.029)	(0.025)
distance + distance × large discrepancy	−0.028***	−0.027	−0.008	0.009	−0.082	−0.082	−0.010	−0.035
	(0.000)	(0.342)	(0.372)	(0.771)	(0.203)	(0.242)	(0.836)	(0.543)
distance × large discrepancy - distance × small discrepancy	−0.005	−0.001	0.051***	0.138***	0.149	0.200*	−0.142*	−0.189**
	(0.664)	(0.989)	(0.000)	(0.008)	(0.196)	(0.098)	(0.068)	(0.032)

Table S9. Average treatment effects on party preferences, by party-policy congruence. All models include stratum-fixed effects. Standard errors are robust to heteroskedasticity. See section S2.2.2 for a definition of outcomes. The bottom panel reports the linear combination of parameters reported in each row. The p-value associated with the corresponding F-test is reported in parentheses. The models in columns 1, 3, 5, 7 are used to construct Figure 4 in the manuscript. The *distance treatment* decreased absolute preference for one's favorite party for those participants whose party and policy preferences were not congruent (column 1). It decreased relative preference for that party only for those participants whose party and preferences exhibited a small discrepancy (column 3). Those short-run findings also travel to non-attriters (columns 2, 4). The treatment had long-run effects: those participants whose party and preferences exhibited a small discrepancy were less likely to vote for their favorite party (columns 5, 6), and more likely to vote for their second favorite party (columns 7, 8). The *registration* and *distance treatments* had, by and large, no statistically significant effects on party preferences (models 1 to 8). * $p < .1$; ** $p < .05$; *** $p < .01$.

	Pre-election		Post election
	All	Non-attriters	Non-attriters
distance	−0.013	−0.062	−0.068
	(0.022)	(0.077)	(0.079)
Num.Obs.	6913	701	686
R2	0.033	0.173	0.190
Mean DV (Control)	1.257	1.244	1.184
Min. DV	0	0	0
Max. DV	3	3	3

Table S10. Average treatment effect on political knowledge. All models include stratum-fixed effects. Standard errors are robust to heteroskedasticity. See section S2.2.2 for a definition of outcomes. The *distance treatment* had no statistically significant effect on political knowledge. * $p < .1$; ** $p < .05$; *** $p < .01$.

S5 Robustness checks

S5.1 Trinary moderator specification

	Pre-election		Post-election	
	All	Non-attriters	Turnout	Inferred turnout
registration	0.023	−0.057	−0.178	−0.188
	(0.032)	(0.121)	(0.139)	(0.155)
civics	−0.005	0.346***	0.093	0.076
	(0.048)	(0.132)	(0.096)	(0.090)
distance	−0.005	−0.192*	−0.187	−0.151
	(0.026)	(0.099)	(0.122)	(0.135)
registration × conditional	−0.036	0.056	0.145	0.209
	(0.033)	(0.124)	(0.158)	(0.167)
registration × uncond. voter	−0.029	0.050	0.179	0.170
	(0.033)	(0.122)	(0.153)	(0.168)
civics × conditional	0.028	−0.342**	−0.047	−0.030
	(0.048)	(0.138)	(0.134)	(0.129)
civics × uncond. voter	0.002	−0.396***	−0.113	−0.095
	(0.048)	(0.136)	(0.125)	(0.120)
distance × conditional	0.016	0.205**	0.164	0.153
	(0.027)	(0.101)	(0.135)	(0.144)
distance × uncond. voter	−0.001	0.165	0.158	0.138
	(0.027)	(0.102)	(0.132)	(0.144)
Num.Obs.	7521	701	700	700
R2	0.656	0.763	0.377	0.293
Mean DV (Control, uncond. non-voter)	0.164	0.304	0.500	0.696
Mean DV (Control, conditional)	0.633	0.648	0.581	0.683
Mean DV (Control, cond. voter)	0.969	0.990	0.822	0.836
Min. DV	0	0	0	0
Max. DV	1	1	1	1
distance + distance × conditional	0.011*	0.014	−0.023	0.001
	(0.084)	(0.502)	(0.687)	(0.978)
distance + distance × uncond. voter	−0.006	−0.026	−0.029	−0.013
	(0.247)	(0.249)	(0.563)	(0.789)
registration + registration × conditional	−0.013	−0.001	−0.032	0.021
	(0.142)	(0.971)	(0.666)	(0.726)
registration + registration × uncond. voter	−0.006	−0.008	0.001	−0.019
	(0.457)	(0.715)	(0.991)	(0.769)
civics + civics × conditional	0.022**	0.004	0.046	0.046
	(0.011)	(0.914)	(0.617)	(0.622)
civics + civics × uncond. voter	−0.003	−0.050	−0.020	−0.019
	(0.639)	(0.119)	(0.804)	(0.807)

Table S11. Average treatment effects on turnout, by prior vote intention, trinary moderator. This table reproduces Table S8 but splits the unconditional voter category into unconditional non-voters (the reference category) and unconditional voters. Results are robust to this modification: the *distance* and *registration treatments* significantly increased turnout in the short run for conditional voters only (column 1). Non-attriters show somewhat comparable patterns (column 2). Treatments had no significant long-term effects (columns 3 and 4). * $p < .1$; ** $p < .05$; *** $p < .01$.

S5.2 Effect of all treatments on preferences

	Pre-election				Post-election			
	Abs. pref		Rel. pref		Vote for fav.		Vote for 2nd fav.	
	All	Non attr.	All	Non attr.	Sharp	Imputed	Sharp	Imputed
registration	−0.001	0.002	0.004	−0.014	−0.027	0.021	−0.014	0.019
	(0.009)	(0.029)	(0.010)	(0.036)	(0.090)	(0.101)	(0.056)	(0.071)
civics	0.019*	0.103***	0.007	0.021	0.088	0.087	0.049	0.017
	(0.010)	(0.039)	(0.011)	(0.057)	(0.109)	(0.119)	(0.086)	(0.093)
distance	0.008	0.058**	0.005	0.052	−0.049	0.003	−0.070	−0.050
	(0.007)	(0.026)	(0.008)	(0.036)	(0.074)	(0.083)	(0.046)	(0.057)
small discrepancy	−0.010	0.062*	−0.012	0.046	0.250**	0.286**	−0.051	−0.035
	(0.010)	(0.033)	(0.011)	(0.038)	(0.111)	(0.116)	(0.065)	(0.076)
large discrepancy	−0.004	0.058*	−0.007	0.027	0.087	0.175*	0.023	0.055
	(0.009)	(0.029)	(0.010)	(0.034)	(0.081)	(0.091)	(0.059)	(0.073)
distance × small discrepancy	−0.031**	−0.087*	−0.064***	−0.184***	−0.185	−0.287**	0.198***	0.201**
	(0.012)	(0.049)	(0.014)	(0.057)	(0.121)	(0.130)	(0.076)	(0.089)
distance × large discrepancy	−0.035***	−0.089**	−0.012	−0.044	−0.032	−0.088	0.055	0.020
	(0.011)	(0.038)	(0.012)	(0.046)	(0.099)	(0.109)	(0.069)	(0.083)
registration × small discrepancy	0.015	0.054	0.002	0.025	0.054	0.024	0.031	−0.007
	(0.015)	(0.053)	(0.018)	(0.058)	(0.139)	(0.147)	(0.094)	(0.105)
registration × large discrepancy	0.009	−0.024	0.013	0.041	−0.026	−0.199	0.096	0.057
	(0.013)	(0.042)	(0.014)	(0.051)	(0.115)	(0.124)	(0.083)	(0.100)
civics × small discrepancy	−0.034**	−0.145**	−0.033*	−0.137	−0.282*	−0.188	−0.151	−0.132
	(0.015)	(0.062)	(0.017)	(0.087)	(0.158)	(0.184)	(0.116)	(0.131)
civics × large discrepancy	−0.018	−0.153***	−0.019	−0.061	−0.131	−0.211	−0.077	0.012
	(0.013)	(0.052)	(0.015)	(0.061)	(0.130)	(0.142)	(0.108)	(0.122)
prior	−0.064***	−0.057	0.687***	0.689***	0.011	0.036	−0.119*	−0.067
	(0.012)	(0.049)	(0.017)	(0.068)	(0.098)	(0.107)	(0.061)	(0.074)
Num.Obs.	6825	630	6757	625	551	551	551	551
R2	0.637	0.688	0.444	0.572	0.275	0.231	0.207	0.214
Mean DV (Control, no discrepancy)	0.628	0.555	0.116	0.066	0.167	0.278	0.093	0.148
Mean DV (Control, small discrepancy)	0.623	0.629	0.109	0.129	0.556	0.630	0.074	0.111
Mean DV (Control, large discrepancy)	0.620	0.596	0.105	0.083	0.315	0.407	0.130	0.185
Min. DV	0	0	−1	−1	0	0	0	0
Max. DV	1	1	1	1	1	1	1	1
distance + distance × small discrepancy	−0.023**	−0.029	−0.059***	−0.132***	−0.233**	−0.284***	0.128**	0.151**
	(0.018)	(0.472)	(0.000)	(0.003)	(0.016)	(0.005)	(0.031)	(0.027)
distance + distance × large discrepancy	−0.028***	−0.031	−0.008	0.008	−0.081	−0.085	−0.014	−0.030
	(0.001)	(0.287)	(0.376)	(0.799)	(0.209)	(0.220)	(0.782)	(0.603)
registration + registration × small discrepancy	0.013	0.056	0.006	0.010	0.026	0.045	0.016	0.013
	(0.302)	(0.219)	(0.709)	(0.828)	(0.814)	(0.685)	(0.818)	(0.873)
registration + registration × large discrepancy	0.008	−0.022	0.017	0.026	−0.053	−0.178**	0.082	0.076
	(0.425)	(0.531)	(0.121)	(0.538)	(0.513)	(0.038)	(0.207)	(0.298)
civics + civics × small discrepancy	−0.015	−0.041	−0.027*	−0.115	−0.194	−0.101	−0.103	−0.115
	(0.216)	(0.452)	(0.053)	(0.108)	(0.134)	(0.503)	(0.232)	(0.257)
civics + civics × large discrepancy	0.001	−0.050	−0.012	−0.039	−0.043	−0.124	−0.028	0.029
	(0.917)	(0.241)	(0.286)	(0.297)	(0.627)	(0.202)	(0.705)	(0.738)
distance × large discrepancy - distance × small discrepancy	−0.004	−0.002	0.052***	0.140***	0.153	0.199	−0.142*	−0.181**
	(0.725)	(0.967)	(0.000)	(0.007)	(0.187)	(0.101)	(0.065)	(0.039)

Table S12. Average effect of all treatments on party preferences, by party-policy congruence. This table reproduces Table S9 but further controls for assignment to the registration and civics treatments, as well as potential heterogeneous subgroup effects. All models include stratum-fixed effects. Standard errors are robust to heteroskedasticity. See section S2.2.2 for a definition of outcomes. The bottom panel reports the linear combination of parameters reported in each row. The p-value associated with the corresponding F-test is reported in parentheses. The *registration* and *distance treatments* had, by and large, no statistically significant effects on party preferences (models 1 to 8). * $p < .1$; ** $p < .05$; *** $p < .01$.

S5.3 Attrition and power

S5.3.1 Differential attrition

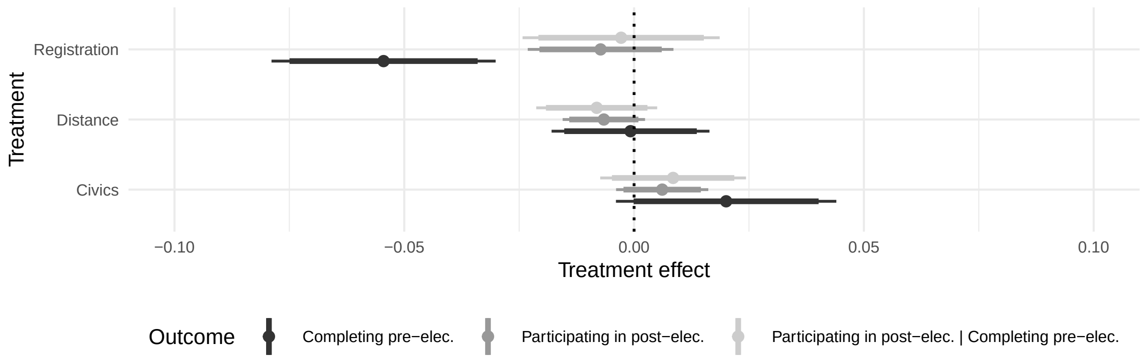

Fig. S8. Effect of treatment assignment on the probability of attrition. We report within-stratum estimates with heteroskedastic-robust standard errors for the unconditional probability of completing the pre-election survey (black) and of participating in the post-election survey (dark gray), as well as the probability of participating in the post-election survey conditional on having completed the pre-election survey (light gray). We use the control condition as a reference category. Bars represent 90 and 95% confidence intervals. Assignment to the *registration treatment* decreased the probability of completing the pre-election survey, but had no impact on the probability of participating in the post-election survey. Assignment to the other treatments had no statistically significant impact on attrition.

S5.3.2 Inverse probability weighted estimates

This section reports inverse probability weighted estimates for our long-run outcomes. Doing so, we correct for potential bias owing to attrition conditional on observables.

To obtain the sampling weights, we consider the sample of those respondents who completed the pre-election survey and model their participation into the post-election survey. Our model uses treatment assignment, as well as all available pre-treatment covariates (See Table S4 for descriptive statistics); that is:

- Age (continuous variable)
- Female (binary variable)
- Education level (categorical variable, reference category: none)
- Marital status (categorical variable, reference category: single)
- Occupation (categorical variable, reference category: student)
- Interest in politics (continuous variable)
- Pre-treatment registration status (categorical variable, reference category: don't know)
- Participation in the 2016 legislative election (binary variable)
- Prior turnout intention (continuous variable)
- Prior level of support for favorite party (continuous variable)
- Prior level of support for second favorite party (continuous variable)
- Favorite party (categorical variable, reference category: PJD)
- Second favorite party (categorical variable, reference category: PJD)

- Discrepancy (categorical variable, reference category: congruent)
- Pre-election survey round (categorical variable, reference category: round 1)

We also include all two-way interactions between those covariates. We select non-zero predictors using the LASSO algorithm, and tune the shrinkage parameter λ through 10-fold cross-validation.

	Turnout		Inferred turnout	
	(1)	(2)	(3)	(4)
registration	−0.051	−0.075	−0.078	−0.099
	(0.063)	(0.065)	(0.062)	(0.063)
civics	−0.021	−0.015	−0.021	−0.015
	(0.080)	(0.080)	(0.080)	(0.080)
distance	−0.044	−0.035	−0.006	−0.002
	(0.048)	(0.049)	(0.046)	(0.048)
registration × conditional	0.017	0.060	0.086	0.098
	(0.102)	(0.104)	(0.090)	(0.096)
civics × conditional	0.046	0.022	0.046	0.022
	(0.128)	(0.127)	(0.128)	(0.127)
distance × conditional	0.049	0.057	0.025	0.032
	(0.077)	(0.079)	(0.071)	(0.075)
Num.Obs.	599	599	599	599
R2	0.379	0.364	0.302	0.288
Mean DV (Control, unconditional)	0.810	0.826	0.823	0.834
Mean DV (Control, conditional)	0.592	0.617	0.690	0.708
Min. DV	0	0	0	0
Max. DV	1	1	1	1
distance + distance × conditional	0.005	0.022	0.019	0.030
	(0.931)	(0.715)	(0.731)	(0.605)
registration + registration × conditional	−0.034	−0.015	0.008	−0.001
	(0.677)	(0.857)	(0.897)	(0.990)
civics + civics × conditional	0.025	0.007	0.024	0.007
	(0.806)	(0.947)	(0.808)	(0.947)
IPW	_	✓	_	✓

Table S13. Inverse probability weighted estimates for turnout. This table reproduces models 3 and 4 in Table S8. The IPW columns (i.e., models 2 and 4) report inverse probability weighted estimates for the sample of those respondents for which the full set of control covariates is available. We also report, for comparison, unweighted estimates (models 1 and 3). Results are similar to those of Table S8: treatments had no significant long-term effects, neither for likely conditional voters, nor for likely unconditional voters and non-voters. * p < .1; ** p < .05; *** p < .01.

	Vote for fav.				Vote for 2nd fav.			
	Sharp		Imputed		Sharp		Imputed	
	(1)	(2)	(3)	(4)	(5)	(6)	(7)	(8)
distance	−0.057	−0.057	0.004	−0.011	−0.047	−0.047	−0.019	−0.014
	(0.077)	(0.083)	(0.086)	(0.092)	(0.047)	(0.046)	(0.057)	(0.056)
small discrepancy	0.187**	0.162*	0.230**	0.189*	−0.052	−0.035	−0.030	−0.016
	(0.093)	(0.095)	(0.095)	(0.098)	(0.041)	(0.040)	(0.053)	(0.050)
large discrepancy	0.031	0.016	0.055	0.028	0.071	0.102*	0.130**	0.166***
	(0.071)	(0.077)	(0.079)	(0.083)	(0.053)	(0.056)	(0.063)	(0.064)
distance × small discrepancy	−0.150	−0.129	−0.263*	−0.223	0.163**	0.167**	0.137	0.135
	(0.126)	(0.132)	(0.134)	(0.140)	(0.078)	(0.076)	(0.088)	(0.084)
distance × large discrepancy	−0.006	−0.006	−0.095	−0.087	0.020	−0.006	−0.050	−0.088
	(0.102)	(0.109)	(0.114)	(0.120)	(0.071)	(0.071)	(0.085)	(0.084)
prior	−0.013	−0.064	0.051	−0.006	−0.142**	−0.141**	−0.066	−0.071
	(0.102)	(0.105)	(0.111)	(0.115)	(0.062)	(0.065)	(0.075)	(0.074)
Num.Obs.	520	520	520	520	520	520	520	520
R2	0.267	0.278	0.223	0.233	0.202	0.225	0.219	0.232
Mean DV (Control, no discrepancy)	0.241	0.264	0.333	0.357	0.093	0.085	0.130	0.113
Mean DV (Control, small discrepancy)	0.510	0.510	0.612	0.609	0.041	0.038	0.082	0.072
Mean DV (Control, large discrepancy)	0.320	0.335	0.398	0.409	0.155	0.176	0.223	0.240
Min. DV	0	0	0	0	0	0	0	0
Max. DV	1	1	1	1	1	1	1	1
distance + distance × small discrepancy	−0.208**	−0.186*	−0.259**	−0.234**	0.116*	0.120**	0.119*	0.120*
	(0.037)	(0.069)	(0.011)	(0.027)	(0.058)	(0.045)	(0.084)	(0.063)
distance + distance × large discrepancy	−0.063	−0.063	−0.091	−0.098	−0.027	−0.053	−0.069	−0.102*
	(0.346)	(0.362)	(0.212)	(0.191)	(0.613)	(0.340)	(0.263)	(0.099)
IPW	_	✓	_	✓	_	✓	_	✓

Table S14. Inverse probability weighted estimates for party preferences. This table reproduces models 4 to 8 in S9. The IPW columns (i.e., even-numbered models) report inverse probability weighted estimates for the sample of those respondents for which the full set of control covariates is available. We also report, for comparison, unweighted estimates (odd-numbered models). Results are similar to those of Table S8: in the long run, those participants whose party and preferences exhibited a small discrepancy were less likely to vote for their favorite party (columns 1 to 4), and more likely to vote for their second favorite party (columns 5 to 8). The *registration* and *distance treatments* had, by and large, no statistically significant effects on party preferences (models 1 to 8). * $p < .1$; ** $p < .05$; *** $p < .01$.

S5.3.3 Power analysis

In this section, we conduct ex-post power analysis, in order to examine the extent to which the uncertainty surrounding our estimates is affected by sample size. Table S15 below reports all of our estimates of interest. For each estimate μ with its associated variance σ^2, we report the associated Hedge's g; that is, the standardized effect size adjusted for unequal sample sizes among treatment and control. We have $g = \frac{\mu}{\sigma_{\text{pooled}}}$, where σ_{pooled} is the outcome's pooled and weighted standard deviation. With $\sigma^2_{y,T}, \sigma^2_{y,C}$ the outcome's variance in the treatment and control groups respectively and n_T, n_C the respective sizes of such groups, the expression for the outcome's pooled and weighted standard deviation is

$$\sigma_{\text{pooled}} = \sqrt{\frac{(n_T - 1)\sigma^2_{y,T} + (n_C - 1)\sigma^2_{y,C}}{n_T + n_C - 2}}$$

We then report the minimum detectable effect (MDE) associated with our estimates. The MDE indicates the smallest effect size that can be detected at the 95% confidence level with 80% power. In other words, it is the smallest effect that appears statistically significant at the 5% confidence level and 80% power. We report the MDE both in the scale of the original variable (MDE $= 2.8 \times \sigma$), as well as the standardized MDE ($\frac{\text{MDE}}{\sigma_{\text{pooled}}}$). The standardized MDE allows comparing with Cohen's rule of thumb to identify the effect sizes that our intervention is powered to detect (.2 = small effect; .5 = medium effect; .8 = large effect).

For each estimate, we report whether it is statistically significant at the 95% confidence level and whether it is statistically equivalent to zero at the 5% significance level. Equivalence tests flip conventional hypothesis testing: instead of positing $\mu = 0$ as the null hypothesis, such tests posit $\mu \neq 0$ as the null. In practice, this requires defining a Region Of Practical Equivalence (ROPE). The ROPE defines an interval of effect sizes that are too small to be considered meaningfully different from zero, with $\mu \notin$ ROPE the test's null hypothesis. Following Kruschke (*57*), we define the ROPE to be half of a small effect (i.e., $g = .1$). That is, in the scale of the original variable, ROPE $= [-.1 \times \sigma, .1 \times \sigma]$. We conduct our equivalence tests using the Two One-Sampled T-test (TOST) procedure (*58*). We reject the null and conclude that μ is statistically equivalent to zero at the 5% confidence level if the 95% confidence interval overlaps with the ROPE, and fail to reject the null otherwise.

Following Lakens (*58*), we finally conclude that μ is different from zero if it is both statistically significant at the 5% confidence level and not equivalent to zero at the 5% confidence level. We conclude that μ is equal to 0 if it is both not statistically significant at the 5% confidence level and equivalent to zero at the 5% confidence level. We conclude that μ is ambiguous otherwise.

Table S15 shows that our short-term effects (i.e., effects estimated using outcomes collected during the pre-election survey) are sufficiently well-powered to detect small effects (i.e., $g < .2$). The estimates that are statistically significant are also not statistically equivalent to zero, while the estimates that are not statistically significant are also statistically equivalent to zero.

Table S15 also shows that our long-term effects (i.e., effects estimated using outcomes collected during the post-election survey) are only powered to detect small to medium (i.e., $g \in [.2, .5]$), or medium to large (i.e., $g \in [.5, .8]$) effects. While the estimates that are statistically significant are also not statistically equivalent to zero, the estimates that are not statistically significant are also too large to be considered statistically equivalent to zero.

Outcome	Horizon	Group	Reference	Treatment	Estimate	MDE	ROPE	Significance	Equivalence	Conclusion
turnout intention	short	all	Table S7, model 1	civics	0.008 (0.025)	0.016 (0.052, small)	0.031	_	✓	0
				distance	0.002 (0.005)	0.012 (0.038, small)	0.031	_	✓	0
				registration	−0.007 (0.021)	0.018 (0.058, small)	0.031	_	✓	0
		unconditional	Table S8, model 1	civics	−0.003 (0.010)	0.022 (0.064, small)	0.031	_	✓	0
				distance	−0.006 (0.018)	0.017 (0.049, small)	0.031	_	✓	0
				registration	−0.001 (0.003)	0.026 (0.077, small)	0.031	_	✓	0
		conditional		civics	0.022 (0.096)	0.024 (0.106, small)	0.031	✓	_	not 0
				distance	0.011 (0.046)	0.017 (0.074, small)	0.031	_	✓	0
				registration	−0.013 (0.056)	0.025 (0.106, small)	0.031	_	✓	0
turnout	long	all	Table S7, model 3	civics	0.012 (0.026)	0.167 (0.359, small - medium)	0.047	_	_	ambiguous
				distance	−0.035 (0.075)	0.101 (0.217, small - medium)	0.047	_	_	ambiguous
				registration	−0.029 (0.062)	0.130 (0.279, small - medium)	0.047	_	_	ambiguous
		unconditional	Table S8, model 3	civics	−0.019 (0.043)	0.216 (0.493, small - medium)	0.047	_	_	ambiguous
				distance	−0.046 (0.104)	0.130 (0.296, small - medium)	0.047	_	_	ambiguous
				registration	−0.026 (0.059)	0.164 (0.375, small - medium)	0.047	_	_	ambiguous
		conditional		civics	0.046 (0.096)	0.260 (0.533, medium - large)	0.047	_	_	ambiguous
				distance	−0.023 (0.048)	0.161 (0.330, small - medium)	0.047	_	_	ambiguous
				registration	−0.032 (0.067)	0.210 (0.431, small - medium)	0.047	_	_	ambiguous
learning	short	all	Table S10, model 1	distance	−0.013 (0.015)	0.060 (0.068, small)	0.090	_	✓	0
	long		Table S10, model 3	distance	−0.068 (0.076)	0.221 (0.247, small - medium)	0.089	_	_	ambiguous
party preference (absolute)	short	no discrepancy	Table S9, model 1	distance	0.008 (0.026)	0.021 (0.067, small)	0.032	_	✓	0
		small discrepancy		distance	−0.022 (0.069)	0.027 (0.085, small)	0.032	✓	_	not 0
		large discrepancy		distance	−0.028 (0.085)	0.022 (0.068, small)	0.032	✓	_	not 0
party preference (relative)		no discrepancy	Table S9, model 3	distance	0.005 (0.017)	0.024 (0.083, small)	0.029	_	✓	0
		small discrepancy		distance	−0.059 (0.195)	0.032 (0.107, small)	0.029	✓	_	not 0
		large discrepancy		distance	−0.008 (0.027)	0.024 (0.085, small)	0.029	_	✓	0
vote choice (fav. party)	long	no discrepancy	Table S9, model 5	distance	−0.037 (0.086)	0.207 (0.489, small - medium)	0.045	_	_	ambiguous
		small discrepancy		distance	−0.231 (0.488)	0.270 (0.571, medium - large)	0.045	✓	_	not 0
		large discrepancy		distance	−0.082 (0.178)	0.179 (0.391, small - medium)	0.045	_	_	ambiguous
vote choice (2nd fav. party)		no discrepancy	Table S9, model 7	distance	−0.062 (0.226)	0.131 (0.478, small - medium)	0.030	_	_	ambiguous
		small discrepancy		distance	0.132 (0.457)	0.169 (0.586, medium - large)	0.030	✓	_	not 0
		large discrepancy		distance	−0.010 (0.032)	0.141 (0.437, small - medium)	0.030	_	_	ambiguous

Table S15. Power analysis. This table examines all effect sizes of interest. Estimates are short- or long-term if they concern outcomes that were measured in the pre- or post-election survey, respectively. The Estimate column reports estimated effects in the original scale, with the associated Hedge's g in parenthesis. The Minimum Detectable Effect (MDE) column reports the MDE at the 95% significance level with 80% power in the original scale, with the associated Hedge's g and effect size category in parenthesis (small: $g < .2$, small-medium: $g \in [.2, .5]$, medium-large: $g \in [.5, .8]$). The ROPE column reports the region of practical equivalence in the original scale. The Significance and Equivalence columns report, respectively, whether significance and equivalence tests at the 95% confidence level are significant (✓) or not (_), and whether it is statistically equivalent to 0 (✓) or not (_). The Conclusion column reports that estimates are "not 0" if they are both significant and not equivalent, "0" if they are not significant and equivalent, and "ambiguous" otherwise. See the discussion in section S5.3.3 for details.

S5.4 Moderator importance

In this section, we report the estimates of the causal forest approach for estimating heterogeneous treatment effects (*21*). We estimate a causal forest for each of our three treatments. Since the *registration treatment* was only administered during round 1, and the *civics treatment* during rounds 2 and 3, we estimate causal forests for the *registration treatment* using only round 1 participants, and causal forests for the *civics treatment* using only rounds 2 and 3 participants. For each causal forest, we use the remaining two treatments as moderators. We also consider all available pre-treatment covariates as moderators. Section S5.3.2 reports all such covariates. As compared to the LASSO approach used for deriving inverse probability weighted estimates, we amend the modeling of two ordinal variables (education and discrepancy), which we now treat as continuous variables.

The plots below report the *variable importance* of each moderator; that is, a weighted sum of the number of times each moderator was split on at each depth in the forest.

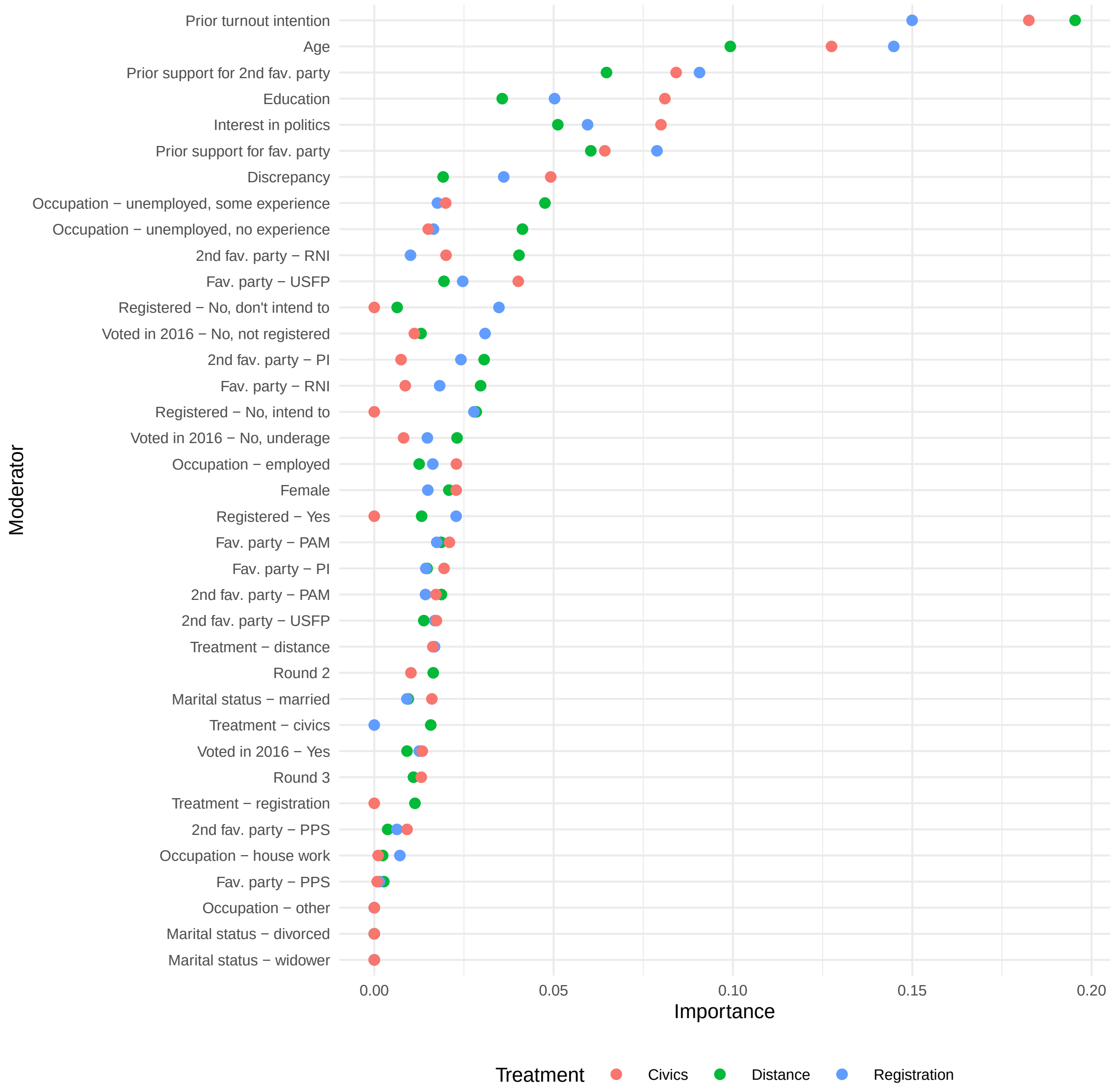

Fig. S9. Moderator importance on turnout intention. We report the importance of a series of potential moderators for each of our three treatments on turnout intention. Prior turnout intention is the most important moderator.

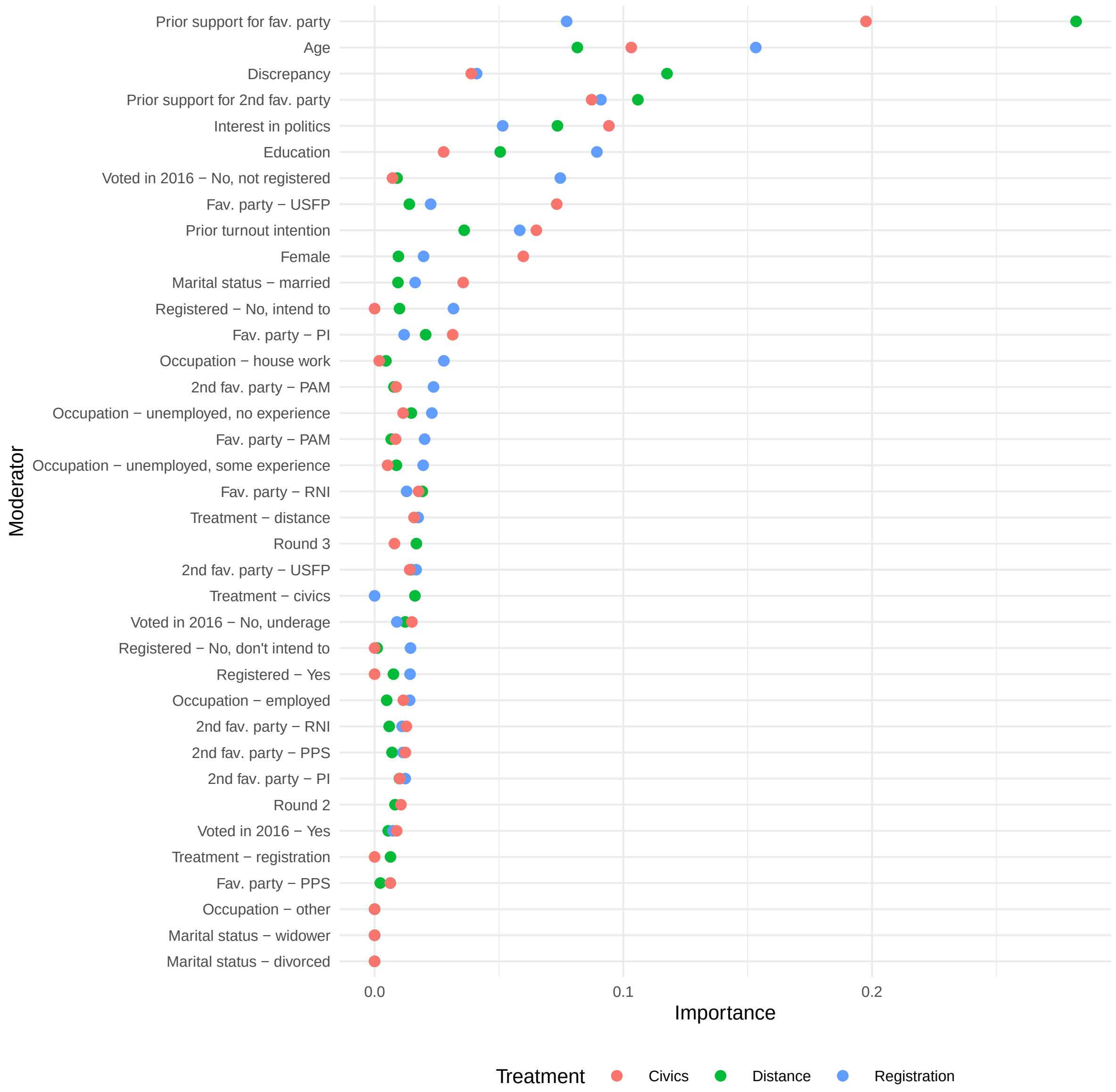

Fig. S10. Moderator importance on party preferences. We report the importance of a series of potential moderators for each of our three treatments on absolute support for one's favorite party. Discrepancy is the second-most important moderator for the *distance treatment*.

S6 Pre-registered tests

This section reports all the tests registered in the pre-analysis plan. All models include stratum-fixed effects, with heteroskedastic-robust standard errors. All tables use the following convention to denote p-value cutoffs: * $p < .1$; ** $p < .05$; *** $p < .01$. The t_1, t_2, t_3 column labels correspond, respectively, to outcomes collected during the modules "posterior 1," "posterior 2," and "posterior 3" described in the survey flow (Figure S1). For each table, we report in the caption when a model was used in the main analysis.

	Pre-election		Post-election		
	t_1	t_2	Registration	Turnout	Inferred turnout
registration	−0.003	−0.007	0.027	−0.029	−0.015
	(0.006)	(0.006)	(0.111)	(0.046)	(0.042)
civics	0.012**	0.008		0.012	0.012
	(0.005)	(0.006)		(0.060)	(0.060)
distance	−0.003	0.002	−0.144	−0.035	−0.014
	(0.004)	(0.004)	(0.112)	(0.036)	(0.034)
Num.Obs.	7521	7521	154	700	700
R2	0.737	0.656	0.402	0.374	0.289
Mean DV (Control)	0.746	0.753	0.551	0.672	0.746
Min. DV	0	0	0	0	0
Max. DV	1	1	1	1	1

Table S16. Hypothesis 1. The models in columns 2, 4, 5 correspond, respectively, to the models in columns 1, 3, 5 in Table S7. The model in column 3 (Registration) is estimated on the set of endline survey participants that were not registered during the baseline survey. Since those participants were only eligible during round 1 of the baseline survey, during which the civics treatment was not administered, the effect of the civics treatment cannot be estimated for those participants.

	Pre-election	Post-election
distance	−0.013	−0.068
	(0.022)	(0.079)
Num.Obs.	6913	686
R2	0.033	0.190
Mean DV (Control)	1.257	1.184
Min. DV	0	0
Max. DV	3	3

Table S17. Hypothesis 2.a. The models in columns 1, 2 correspond, respectively, to the models in columns 1, 3 in Table S10.

	Abs. pref			Rel. pref			Vote for fav.	
	t_1	t_2	t_3	t_1	t_2	t_3	Sharp	Imputed
distance	0.005	0.008	0.051	0.003	0.005	0.048	−0.036	0.014
	(0.006)	(0.007)	(0.055)	(0.007)	(0.008)	(0.074)	(0.074)	(0.083)
discrepancy	0.002	−0.011*	0.106**	0.004	−0.013*	0.128**	0.111*	0.138**
	(0.006)	(0.006)	(0.044)	(0.006)	(0.007)	(0.059)	(0.064)	(0.069)
distance × discrepancy	−0.004	−0.034***	−0.146**	−0.015*	−0.032***	−0.138	−0.094	−0.160
	(0.008)	(0.010)	(0.068)	(0.009)	(0.011)	(0.091)	(0.092)	(0.102)
prior	−0.073***	−0.065***	0.084	0.755***	0.683***	0.338***	0.011	0.048
	(0.012)	(0.012)	(0.069)	(0.015)	(0.017)	(0.100)	(0.096)	(0.106)
Num.Obs.	6821	6809	637	6763	6741	625	551	551
R2	0.749	0.635	0.228	0.583	0.440	0.197	0.260	0.211
Mean DV (Control, no discrepancy)	0.625	0.642	0.477	0.120	0.129	0.011	0.226	0.313
Mean DV (Control, discrepancy)	0.618	0.623	0.600	0.116	0.109	0.160	0.374	0.460
Min. DV	0	0	0	−1	−1	−1	0	0
Max. DV	1	1	1	1	1	1	1	1
distance + distance × discrepancy	0.001	−0.026***	−0.095**	−0.011**	−0.027***	−0.090*	−0.129**	−0.146**
	(0.860)	(0.000)	(0.013)	(0.044)	(0.000)	(0.071)	(0.017)	(0.012)

Table S18. Hypothesis 2.b, discrete shock

	Abs. pref			Rel. pref			Vote for fav.	
	t_1	t_2	t_3	t_1	t_2	t_3	Sharp	Imputed
distance	0.002	−0.014***	−0.050	−0.006	−0.017***	−0.053	−0.104**	−0.102**
	(0.004)	(0.005)	(0.031)	(0.004)	(0.005)	(0.040)	(0.043)	(0.046)
discrepancy	0.004	−0.006	0.186***	0.002	−0.014	0.311***	0.270***	0.373***
	(0.008)	(0.008)	(0.064)	(0.008)	(0.009)	(0.077)	(0.086)	(0.091)
distance × discrepancy	−0.008	−0.054***	−0.186*	−0.015	−0.093***	−0.292**	−0.294**	−0.369***
	(0.010)	(0.013)	(0.097)	(0.012)	(0.016)	(0.124)	(0.122)	(0.132)
prior	−0.073***	−0.063***	0.086	0.755***	0.686***	0.337***	0.011	0.052
	(0.012)	(0.012)	(0.069)	(0.015)	(0.017)	(0.100)	(0.097)	(0.106)
Num.Obs.	6821	6809	637	6763	6741	625	551	551
R2	0.749	0.635	0.231	0.583	0.445	0.213	0.272	0.232
Mean DV (Control)	0.620	0.630	0.550	0.117	0.116	0.099	0.313	0.399
Min. DV	0	0	0	−1	−1	−1	0	0
Max. DV	1	1	1	1	1	1	1	1

Table S19. Hypothesis 2.b, continuous shock

	Pre-election	Post-election
distance	−0.002	−0.092
	(0.031)	(0.106)
registration or civics	−0.003	−0.031
	(0.030)	(0.109)
[registration or civics] × distance	−0.023	0.050
	(0.043)	(0.161)
Num.Obs.	6913	686
R2	0.033	0.190
Mean DV (Control, no registration nor civics)	1.258	1.207
Mean DV (Control, registration or civics)	1.256	1.163
Min. DV	0	0
Max. DV	3	3
distance + [registration or civics] × distance	−0.025	−0.042
	(0.412)	(0.723)

Table S20. Hypothesis 3.a

	Abs. pref			Rel. pref			Vote for fav.	
	t_1	t_2	t_3	t_1	t_2	t_3	Sharp	Imputed
distance	0.0002	0.009	0.125	−0.006	0.006	0.160	0.091	0.095
	(0.009)	(0.010)	(0.080)	(0.010)	(0.012)	(0.107)	(0.116)	(0.127)
registration or civics	0.007	0.011	0.112*	0.005	0.006	0.166*	0.101	0.104
	(0.009)	(0.010)	(0.066)	(0.010)	(0.011)	(0.087)	(0.094)	(0.105)
discrepancy	0.009	−0.009	0.218***	0.016*	−0.012	0.249***	0.212**	0.231**
	(0.008)	(0.009)	(0.061)	(0.009)	(0.010)	(0.079)	(0.086)	(0.097)
distance × discrepancy	−0.004	−0.028**	−0.258***	−0.012	−0.027*	−0.246*	−0.262*	−0.215
	(0.011)	(0.013)	(0.096)	(0.013)	(0.015)	(0.129)	(0.138)	(0.154)
[registration or civics] × distance	0.009	−0.003	−0.143	0.017	−0.002	−0.218	−0.227	−0.155
	(0.013)	(0.015)	(0.110)	(0.014)	(0.017)	(0.151)	(0.150)	(0.169)
[registration or civics] × discrepancy	−0.012	−0.002	−0.221**	−0.023*	−0.003	−0.240**	−0.196	−0.183
	(0.012)	(0.012)	(0.085)	(0.013)	(0.014)	(0.114)	(0.123)	(0.137)
[registration or civics] × distance × discrepancy	0.0004	−0.012	0.215	−0.003	−0.011	0.209	0.307*	0.093
	(0.016)	(0.019)	(0.132)	(0.018)	(0.022)	(0.179)	(0.182)	(0.206)
prior	−0.073***	−0.065***	0.078	0.755***	0.683***	0.332***	0.004	0.036
	(0.012)	(0.012)	(0.069)	(0.015)	(0.017)	(0.101)	(0.098)	(0.107)
Num.Obs.	6821	6809	637	6763	6741	625	551	551
R2	0.750	0.636	0.239	0.584	0.440	0.208	0.268	0.221
Mean DV (Control, no discrepancy)	0.625	0.642	0.477	0.120	0.129	0.011	0.226	0.313
Mean DV (Control, discrepancy)	0.618	0.623	0.600	0.116	0.109	0.160	0.374	0.460
Min. DV	0	0	0	−1	−1	−1	0	0
Max. DV	1	1	1	1	1	1	1	1
distance + distance × discrepancy	−0.004	−0.019**	−0.133**	−0.018**	−0.021**	−0.086	−0.172**	−0.120
	(0.571)	(0.024)	(0.011)	(0.013)	(0.024)	(0.195)	(0.025)	(0.151)
[registration or civics] × distance + [registration or civics] × distance × discrepancy	0.009	−0.015	0.072	0.014	−0.013	−0.008	0.081	−0.062
	(0.355)	(0.241)	(0.341)	(0.206)	(0.353)	(0.933)	(0.445)	(0.589)

Table S21. Hypothesis 3.b, discrete shock

	Abs. pref			Rel. pref			Vote for fav.	
	t_1	t_2	t_3	t_1	t_2	t_3	Sharp	Imputed
distance	−0.002	−0.010	−0.047	−0.014**	−0.013*	−0.009	−0.100	−0.059
	(0.005)	(0.007)	(0.043)	(0.006)	(0.007)	(0.055)	(0.063)	(0.068)
registration or civics	−0.0008	0.010	−0.026	−0.010*	0.004	0.021	−0.035	−0.017
	(0.006)	(0.006)	(0.042)	(0.006)	(0.007)	(0.056)	(0.063)	(0.067)
discrepancy	0.012	−0.001	0.219**	0.015	−0.010	0.313***	0.386***	0.431***
	(0.010)	(0.011)	(0.086)	(0.012)	(0.013)	(0.092)	(0.124)	(0.130)
distance × discrepancy	−0.002	−0.050***	−0.213	−0.015	−0.084***	−0.324*	−0.402**	−0.456**
	(0.014)	(0.017)	(0.131)	(0.016)	(0.022)	(0.167)	(0.184)	(0.200)
[registration or civics] × distance	0.009	−0.009	−0.006	0.016*	−0.008	−0.091	−0.010	−0.090
	(0.008)	(0.010)	(0.063)	(0.009)	(0.011)	(0.082)	(0.086)	(0.093)
[registration or civics] × discrepancy	−0.016	−0.010	−0.071	−0.026	−0.007	−0.007	−0.249	−0.128
	(0.015)	(0.016)	(0.129)	(0.017)	(0.018)	(0.163)	(0.171)	(0.183)
[registration or civics] × distance × discrepancy	−0.012	−0.008	0.060	0.0002	−0.017	0.075	0.224	0.174
	(0.021)	(0.027)	(0.188)	(0.023)	(0.032)	(0.250)	(0.251)	(0.273)
prior	−0.073***	−0.063***	0.083	0.755***	0.686***	0.335***	0.007	0.044
	(0.012)	(0.012)	(0.069)	(0.015)	(0.017)	(0.100)	(0.097)	(0.106)
Num.Obs.	6821	6809	637	6763	6741	625	551	551
R2	0.750	0.635	0.233	0.584	0.445	0.216	0.277	0.238
Mean DV (Control)	0.620	0.630	0.550	0.117	0.116	0.099	0.313	0.399
Min. DV	0	0	0	−1	−1	−1	0	0
Max. DV	1	1	1	1	1	1	1	1

Table S22. Hypothesis 3.b, continuous shock

	Pre-election	Post-election
distance	0.013	0.118
	(0.044)	(0.149)
late	−0.031	0.115
	(0.042)	(0.152)
distance × late	−0.020	−0.152
	(0.060)	(0.256)
Num.Obs.	3614	254
R2	0.013	0.057
Mean DV (Control, early)	1.271	1.024
Mean DV (Control, late)	1.243	1.109
Min. DV	0	0
Max. DV	3	3
distance + distance × late	−0.007	−0.034
	(0.866)	(0.860)

Table S23. Hypothesis 4.a

	Abs. pref			Rel. pref			Vote for fav.	
	t_1	t_2	t_3	t_1	t_2	t_3	Sharp	Imputed
distance	0.011	0.018	0.017	0.011	0.006	0.004	0.063	−0.056
	(0.012)	(0.014)	(0.106)	(0.013)	(0.017)	(0.156)	(0.133)	(0.152)
discrepancy	0.003	−0.011	0.149*	0.006	−0.025*	0.176	0.097	0.072
	(0.011)	(0.011)	(0.083)	(0.013)	(0.014)	(0.116)	(0.122)	(0.133)
distance × discrepancy	−0.019	−0.046**	−0.197	−0.033*	−0.022	−0.200	−0.188	−0.105
	(0.015)	(0.018)	(0.127)	(0.018)	(0.022)	(0.173)	(0.168)	(0.187)
prior	−0.053***	−0.032*	0.053	0.768***	0.699***	0.247*	0.112	0.158
	(0.015)	(0.016)	(0.103)	(0.020)	(0.023)	(0.139)	(0.133)	(0.155)
late	−0.004	0.003	−0.014	0.008	−0.003	0.005	0.023	−0.055
	(0.012)	(0.013)	(0.114)	(0.014)	(0.016)	(0.134)	(0.137)	(0.153)
distance × late	0.001	−0.002	0.113	−0.008	0.023	−0.037	−0.055	0.056
	(0.018)	(0.021)	(0.162)	(0.019)	(0.025)	(0.223)	(0.204)	(0.226)
late × discrepancy	0.010	0.007	−0.107	0.003	0.025	−0.213	−0.096	−0.041
	(0.016)	(0.017)	(0.137)	(0.018)	(0.020)	(0.164)	(0.177)	(0.198)
distance × late × discrepancy	0.003	−0.019	−0.087	0.016	−0.047	0.127	0.158	0.025
	(0.023)	(0.027)	(0.200)	(0.025)	(0.031)	(0.267)	(0.247)	(0.275)
Num.Obs.	3585	3579	241	3555	3541	235	211	211
R2	0.732	0.608	0.165	0.578	0.424	0.167	0.119	0.125
Mean DV (Control, no discrepancy)	0.652	0.670	0.484	0.120	0.127	0.054	0.195	0.293
Mean DV (Control, discrepancy)	0.646	0.648	0.593	0.126	0.115	0.152	0.274	0.384
Min. DV	0	0	0	−1	−1	−1	0	0
Max. DV	1	1	1	1	1	1	1	1
distance + distance × discrepancy	−0.008	−0.028**	−0.180***	−0.022*	−0.015	−0.196**	−0.125	−0.161
	(0.414)	(0.016)	(0.010)	(0.059)	(0.249)	(0.012)	(0.208)	(0.127)
distance × late + distance × late × discrepancy	0.004	−0.021	0.026	0.008	−0.024	0.090	0.103	0.081
	(0.783)	(0.225)	(0.835)	(0.608)	(0.209)	(0.560)	(0.508)	(0.637)

Table S24. Hypothesis 4.b, discrete shock

	Abs. pref			Rel. pref			Vote for fav.	
	t_1	t_2	t_3	t_1	t_2	t_3	Sharp	Imputed
distance	−0.001	−0.010	−0.116**	−0.010	−0.008	−0.129*	−0.066	−0.127
	(0.008)	(0.009)	(0.057)	(0.009)	(0.010)	(0.071)	(0.078)	(0.086)
discrepancy	0.016	−0.012	0.244*	0.013	−0.011	0.401***	0.128	0.194
	(0.018)	(0.016)	(0.131)	(0.019)	(0.018)	(0.135)	(0.164)	(0.176)
distance × discrepancy	−0.017	−0.046*	−0.150	−0.050**	−0.095***	−0.286	−0.262	−0.214
	(0.022)	(0.026)	(0.175)	(0.024)	(0.031)	(0.191)	(0.210)	(0.234)
prior	−0.053***	−0.032*	0.071	0.769***	0.701***	0.237	0.149	0.177
	(0.015)	(0.016)	(0.107)	(0.020)	(0.023)	(0.150)	(0.132)	(0.154)
late	0.002	0.008	−0.071	0.010	0.013	−0.097	−0.019	−0.035
	(0.008)	(0.008)	(0.062)	(0.009)	(0.009)	(0.074)	(0.094)	(0.105)
distance × late	0.001	−0.019	0.023	0.001	−0.012	0.021	0.002	−0.009
	(0.011)	(0.014)	(0.106)	(0.012)	(0.015)	(0.133)	(0.136)	(0.149)
late × discrepancy	0.007	0.026	−0.049	−0.009	0.016	−0.084	0.071	0.202
	(0.023)	(0.023)	(0.183)	(0.024)	(0.025)	(0.214)	(0.252)	(0.293)
distance × late × discrepancy	−0.027	−0.057	−0.241	0.025	−0.066	0.040	−0.115	−0.387
	(0.030)	(0.038)	(0.277)	(0.033)	(0.043)	(0.352)	(0.346)	(0.389)
Num.Obs.	3585	3579	241	3555	3541	235	211	211
R2	0.732	0.607	0.164	0.578	0.432	0.193	0.125	0.143
Mean DV (Control)	0.648	0.656	0.553	0.124	0.119	0.116	0.246	0.351
Min. DV	0	0	0	−1	−1	−1	0	0
Max. DV	1	1	1	1	1	1	1	1

Table S25. Hypothesis 4.b, continuous shock

	Pre-election			Post-election							
	GOTV	Redirect	Donate	MP Rep.	Instit. action	Noninstit. action	Efficacy	Interest	News	Donate
registration	−0.029**	−0.023	−0.003	0.039	−0.011	−0.048	−0.046	0.001	−0.030	−0.001
	(0.015)	(0.016)	(0.009)	(0.038)	(0.058)	(0.052)	(0.045)	(0.034)	(0.028)	(0.033)
civics	0.022	0.010	−0.004	0.055	−0.034	−0.025	0.033	0.012	−0.019	0.022
	(0.014)	(0.016)	(0.008)	(0.046)	(0.077)	(0.063)	(0.050)	(0.041)	(0.034)	(0.044)
distance	−0.009	−0.013	0.009	0.025	−0.027	−0.007	0.034	−0.008	0.006	0.050*
	(0.010)	(0.011)	(0.006)	(0.029)	(0.045)	(0.040)	(0.033)	(0.026)	(0.021)	(0.025)
Num.Obs.	7519	7519	7365	596	639	639	641	701	689	684
R2	0.113	0.055	0.023	0.241	0.244	0.220	0.213	0.285	0.183	0.233
Mean DV (Control)	0.707	0.461	0.070	0.425	0.475	0.259	0.533	0.519	0.015	0.087
Min. DV	0	0	0	0	0	0	0	0	−0.554 564 907 275 321	0
Max. DV	1	1	1	1	1	1	1	1	0.445 435 092 724 679	1

Table S26. Hypothesis 5. The pre-registered “Share” outcome is omitted because it was mismeasured.

REFERENCES AND NOTES

1. K. Munger, *Generation Gap: Why the Baby Boomers Still Dominate American Politics and Culture* (Columbia Univ. Press, 2022).

2. W. A. Galston, Civic education and political participation. *PS Polit. Sci. Polit.* **37**, 263–266 (2004).

3. A. L. Campbell, *How Policies Make Citizens: Senior Political Activism and the American Welfare State* (Princeton Univ. Press, 2011).

4. J. B. Holbein, D. S. Hillygus, *Making Young Voters: Converting Civic Attitudes into Civic Action* (Cambridge Univ. Press, 2020).

5. M. G. de Paredes, T. Desrues, Unravelling the adoption of youth quotas in African hybrid regimes: Evidence from Morocco. *J. Mod. Afr. Stud.* **59**, 41–58 (2021), .

6. R. Bourqia, M. Harras, D. Bensaid, *Jeunesse estudiantine marocaine: Valeurs et stratégies* (Faculté des lettres et des sciences humaines de Rabat, 1995).

7. S. Zerhouni, Jeunes et participation politique au maroc, *Tech. Rep.*, Royal Institute for Strategic Studies (2009).

8. M. Manion, The electoral connection in the chinese countryside. *Am. Polit. Sci. Rev.* **90**, 736–748 (1996).

9. T. Shi, Voting and nonvoting in china: Voting behavior in plebiscitary and limited-choice elections. *J. Theor. Polit.* **61**, 1115–1139 (1999).

10. A. Rozenas, Office insecurity and electoral manipulation. *J. Theor. Polit.* **78**, 232–248 (2016).

11. J. Gandhi, Political economy, in *Political Institutions under Dictatorship* (Cambridge Univ. Press, 2009).

12. S. Gehlbach, P. Keefer, Private investment and the institutionalization of collective action in autocracies: Ruling parties and legislatures. *J. Theor. Polit.* **74**, 621–635 (2012).

13. J. Wright, Do authoritarian institutions constrain? how legislatures affect economic growth and investment. *Am. J. Polit. Sci.* **52**, 322–343 (2008).

14. A. Downs, *An Economic Theory of Democracy* (Harper & Row, 1957).

15. R. Ferrali, G. Grossman, M. R. Platas, J. Rodden, Who registers? Village networks, household dynamics, and voter registration in rural Uganda. *Comp. Polit. Stud.* **55**, 899–932 (2022).

16. N. Fuchs-Schündeln, M. Schündeln, Political economy. On the endogeneity of political preferences: Evidence from individual experience with democracy. *Science* **347**, 1145–1148 (2015).

17. F. House, *Freedom in the World 2022: Morocco* (2022).

18. T. Desrues, Authoritarian resilience and democratic representation in Morocco: Royal interference and political parties' leaderships since the 2016 elections. *Mediterr. Politics* **25**, 254–262 (2020).

19. D. Maghraoui, On the relevance or irrelevance of political parties in Morocco. *J. North African Stud.* **25**, 939–959 (2020).

20. We Are Social & Hootsuite, Digital 2021: Morocco, *Tech. Rep.* (2021).

21. S. Athey, J. Tibshirani, S. Wager, Generalized random forests. *Ann. Stat.* **47**, 1148–178 (2019).

22. M. Rolfe, *Voter Turnout: A Social Theory of Political Participation* (Cambridge Univ. Press, 2012).

23. B. Sinclair, *The Social Citizen: Peer Networks and Political Behavior* (University of Chicago Press, 2012).

24. D. A. Siegel, Social networks and collective action. *Am. J. Pol. Sci.* **53**, 122–138 (2009).

25. J. A. Harris, C. Kamindo, P. Van der Windt, Electoral administration in fledgling democracies: Experimental evidence from Kenya. *J. Theor. Polit.* **83**, 947–960 (2021).

26. H. Larreguy, J. Marshall, P. Querubín, Parties, brokers, and voter mobilization: How turnout buying depends upon the party's capacity to monitor brokers. *Am. Polit. Sci. Rev.* **110**, 160–179 (2016).

27. Z. Kunda, The case for motivated reasoning. *Psychol. Bull.* **108**, 480–498 (1990).

28. R. Bayes, J. N. Druckman, Motivated reasoning and climate change. *Curr. Opin. Behav. Sci.* **42**, 27–35 (2021).

29. B. Nyhan, J. Reifler, When corrections fail: The persistence of political misperceptions. *Polit. Behav.* **32**, 303–330 (2010).

30. I.-H. Cheng, A. Hsiaw, Distrust in experts and the origins of disagreement. *J. Econ. Theory* **200**, 105401 (2022).

31. D. E. Campbell, What social scientists have learned about civic education: A review of the literature. *Peabody J. Educ.* **94**, 32–47 (2019).

32. S. Donbavand, B. Hoskins, Citizenship education for political engagement: A systematic review of controlled trials. *Soc. Sci.* **10**, 151 (2021).

33. S. E. Finkel, Can democracy be taught?. *J. Democr.* **14**, 137–151 (2003).

34. S. E. Finkel, A. E. Smith, Civic education, political discussion, and the social transmission of democratic knowledge and values in a new democracy: Kenya 2002. *Am. J. Polit. Sci.* **55**, 417–435 (2011).

35. S. D. Hyde, E. Lamb, O. Samet, *Comp. Pol. Stud.,* in press (2022).

36. E. Mvukiyehe, C. Samii, Promoting democracy in fragile states: Field experimental evidence from liberia. *World Dev.* **95**, 254–267 (2017).

37. M. Lodge, C. S. Taber, in *Elements of Reason: Understanding and Expanding the Limits of Political Rationality*, A. Lupia, M. D. McCubbins, S. L. Popkin, Eds. (Cambridge Univ. Press, 2000), pp. 181–245.

38. D. P. Redlawsk, Hot cognition or cool consideration? Testing the effects of motivated reasoning on political decision making. *J. Polit.* **64**, 1021–1044 (2002).

39. C. S. Taber, M. Lodge, Motivated skepticism in the evaluation of political beliefs. *Am. J. Polit. Sci.* **50**, 755–769 (2006).

40. C. Taber, D. Cann, S. Kucsova, The motivated processing of political arguments. *Polit. Behav.* **31**, 137–155 (2009).

41. C. Adida, J. Gottlieb, E. Kramon, G. McClendon, Reducing or reinforcing in-group preferences? An experiment on information and ethnic voting. *Quart. J. Polit. Sci.* **12**, 437–477 (2017).

42. R. Benabou, J. Tirole, Mindful economics: The production, consumption, and value of beliefs. *J. Economic Perspect.* **30**, 141–164 (2016).

43. S. Finkel, A. Neundorf, E. G. Rascon Ramirez, Can online civic education induce democratic citizenship? Experimental evidence from a new democracy. *Am. J. Polit. Sci.*, 1–18 (2023).

44. B. Marx, V. Pons, T. Suri, Voter mobilisation and trust in electoral institutions: Evidence from Kenya. *Econ. J.* **131**, 2585–2612 (2021).

45. A. Azelton, B. Barrowman, L. Reppell, *Raising their Voices: How Effective Are Pro-Youth Laws and Policies* (CEPPS, 2019).

46. A. Erlich, D. F. Jung, J. D. Long, C. McIntosh, The double-edged sword of mobilizing citizens via mobile phone in developing countries. *Development Engineering* **3**, 34–46 (2018).

47. F. Foos, L. Kostadinov, N. Marinov, F. Schimmelfennig, Does social media promote civic activism? A field experiment with a civic campaign. *Polit. Sci. Res. Methods* **9**, 500–518 (2021).

48. E. Lust-Okar, Elections under authoritarianism: Preliminary lessons from Jordan. *Democratization* **13**, 456–471 (2006).

49. T. Masoud, Are they democrats?: Does it matter? *J. Democr.* **19**, 19–24 (2008).

50. K. Croke, G. Grossman, H. A. Larreguy, J. Marshall, Deliberate disengagement: How education can decrease political participation in electoral authoritarian regimes. *Am. Polit. Sci. Rev.* **110**, 579–600 (2016).

51. H. Larreguy, J. Marshall, The effect of education on civic and political engagement in nonconsolidated democracies: Evidence from Nigeria. *Rev. Econ. Stat.* **99**, 387–401 (2017).

52. A. L. Robinson, J. Gottlieb, How to close the gender gap in political participation: Lessons from matrilineal societies in Africa. *Br. J. Polit. Sci.* **51**, 68–92 (2021).

53. A. M. Guess, M. Lerner, B. Lyons, J. M. Montgomery, B. Nyhan, J. Reifler, N. Sircar, A digital media literacy intervention increases discernment between mainstream and false news in the United States and India. *Proc. Natl. Acad. Sci. U.S.A.* **117**, 15536–15545 (2020).

54. P. M. Ayoub, D. Page, S. Whitt, Pride amid prejudice: The influence of LGBT+ rights activism in a socially conservative society. *Am. Polit. Sci. Rev.* **115**, 467–485 (2021).

55. M. Bennani-Chraïbi, *Partis politiques et protestations au Maroc (1934–2020)* (Presses universitaires de Rennes, 2021).

56. G. Pennycook, Z. Epstein, M. Mosleh, A. Arechar, D. Eckles, D. G. Rand, Shifting attention to accuracy can reduce misinformation online. *Nature* **592**, 590–595 (2021).

57. J. K. Kruschke, Rejecting or accepting parameter values in bayesian estimation *Adv. Meth. Pract. Psychol. Sci.* **1**, 270–280 (2018).

58. D. Lakens, Equivalence tests: A practical primer for *t* tests, correlations, and meta-analyses. *Soc. Psychol. Personal. Sci.* **8**, 355–362 (2017).
